# The G2019S variant of leucine-rich repeat kinase 2 (LRRK2) alters endolysosomal trafficking by impairing the function of the GTPase RAB8A

**DOI:** 10.1074/jbc.RA118.005008

**Published:** 2019-02-01

**Authors:** Pilar Rivero-Ríos, María Romo-Lozano, Jesús Madero-Pérez, Andrew P. Thomas, Alice Biosa, Elisa Greggio, Sabine Hilfiker

**Affiliations:** From the ‡ Institute of Parasitology and Biomedicine “López-Neyra,” Consejo Superior de Investigaciones Científicas (CSIC), Avda del Conocimiento s/n, 18016 Granada, Spain,; the § Department of Pharmacology, Physiology and Neuroscience, New Jersey Medical School, Rutgers, The State University of New Jersey, Newark, New Jersey 07103, and; the ¶ Department of Biology, University of Padova, Padova 35121, Italy

**Keywords:** leucine-rich repeat kinase 2 (LRRK2), Rab, receptor endocytosis, lysosome, receptor recycling, GTPase, neurodegeneration, Parkinson disease, early recycling compartment, endolysosome, protein homeostasis, RAB7A, RAB8A

## Abstract

Mutations in the gene encoding for leucine-rich repeat kinase 2 (LRRK2) are a common cause of hereditary Parkinson's disease. LRRK2 regulates various intracellular vesicular trafficking pathways, including endolysosomal degradative events such as epidermal growth factor receptor (EGFR) degradation. Recent studies have revealed that a subset of RAB proteins involved in secretory and endocytic recycling are LRRK2 kinase substrates *in vivo*. However, the effects of LRRK2-mediated phosphorylation of these substrates on membrane trafficking remain unknown. Here, using an array of immunofluorescence and pulldown assays, we report that expression of active or phosphodeficient RAB8A variants rescues the G2019S LRRK2–mediated effects on endolysosomal membrane trafficking. Similarly, up-regulation of the RAB11–Rabin8–RAB8A cascade, which activates RAB8A, also reverted these trafficking deficits. Loss of RAB8A mimicked the effects of G2019S LRRK2 on endolysosomal trafficking and decreased RAB7A activity. Expression of pathogenic G2019S LRRK2 or loss of RAB8A interfered with EGFR degradation by causing its accumulation in a RAB4-positive endocytic compartment, which was accompanied by a deficit in EGFR recycling and was rescued upon expression of active RAB7A. Dominant-negative RAB7A expression resulted in similar deficits in EGF degradation, accumulation in a RAB4 compartment, and deficits in EGFR recycling, which were all rescued upon expression of active RAB8A. Taken together, these findings suggest that, by impairing RAB8A function, pathogenic G2019S LRRK2 deregulates endolysosomal transport and endocytic recycling events.

## Introduction

Mutations in the gene encoding for leucine-rich repeat kinase 2 (LRRK2)^3^ are the most common cause of familial Parkinson's disease (PD) and are also found in some sporadic cases (1–3). LRRK2 is a large protein harboring a kinase domain, a GTPase domain, and various protein–protein interaction domains. The most prominent variant is G2019S, located in the kinase domain (4). This variant causes an increase in kinase activity that correlates with enhanced neuronal toxicity *in vivo* (5–10). However, despite the importance of LRRK2 for the pathogenesis of PD, the mechanism(s) by which pathogenic G2019S LRRK2 causes neurodegeneration remains unknown.

LRRK2 is widely expressed, suggesting that it may play cellular role(s) common to both neuronal and nonneuronal cell types (11). LRRK2 regulates various vesicular trafficking events, including the endocytic pathway, autophagy/lysosomal pathway, and retromer-mediated pathway between late endosomes and the trans-Golgi network, respectively (12–24). In some cases, those trafficking deficits have been reported to be reversed by either genetic (12) or pharmacological kinase inhibition (21). Our previous studies revealed that G2019S LRRK2 causes endolysosomal trafficking deficits as measured by following the degradative trafficking of the epidermal growth factor receptor (EGFR). Such trafficking deficits were reverted by various kinase inhibitors, correlated with a decrease in RAB7A activity, and could be rescued upon active RAB7A expression (21). Because RAB7A is a crucial regulator of endolysosomal trafficking pathways (25), an LRRK2-mediated deficit in its activity may explain the observed endolysosomal defects.

A recent large-scale phosphoproteomics study has identified a subset of RAB proteins as *in vivo* LRRK2 kinase substrates, with RAB8A being one of the most prominent (25). Phosphomimetic RAB8A variants display impaired interaction with GDP dissociation inhibitor 1/2 (GDI1/2), which is essential to target/extract the protein from the membrane, and with its guanine nucleotide exchange factor (GEF) Rabin8, which is required to activate the protein (25, 26). These biochemical studies led to the proposal that LRRK2-mediated phosphorylation of RAB8A may cause its inactivation (25). However, the cellular consequences with respect to intracellular membrane trafficking events remain unknown.

RAB8A is localized to the Golgi as well as to a tubular early recycling compartment and is known to regulate post-Golgi exocytic membrane trafficking, retromer-mediated trafficking, and endocytic recycling steps (27–30). Recent data suggest that RAB8A may also modulate endolysosomal vesicular trafficking events (31). We therefore sought to determine a possible link between alterations in RAB8A and the endolysosomal degradative trafficking steps that are impaired by G2019S LRRK2.

## Results

### LRRK2 phosphorylates RAB8A but not RAB7A

Because the phosphorylation of RAB8A has been suggested to cause its inactivation (25), we wondered whether pathogenic LRRK2 may cause the reported decrease in RAB7A activity (21) via direct phosphorylation. When comparing the phosphorylation of different RAB proteins *in vitro*, RAB8A was found to serve as an efficient LRRK2 kinase substrate (Fig. S1, *A–C*). In addition, and as described previously (25), phosphorylation of RAB8A was increased with the pathogenic G2019S variant as compared with WT LRRK2 and was largely abolished when mutating the identified LRRK2 phosphorylation site (T72A) (Fig. S1C). In contrast, other RAB proteins tested, including RAB7A, were not phosphorylated to a significant degree (Fig. S1B). Therefore, the previously reported LRRK2-mediated deficit in endolysosomal trafficking (24) does not seem to be due to direct LRRK2-mediated phosphorylation and concomitant inactivation of RAB7A.

### G2019S LRRK2–mediated endolysosomal trafficking deficits are rescued by a RAB11-Rabin8-RAB8A cascade

**Figure 1.**
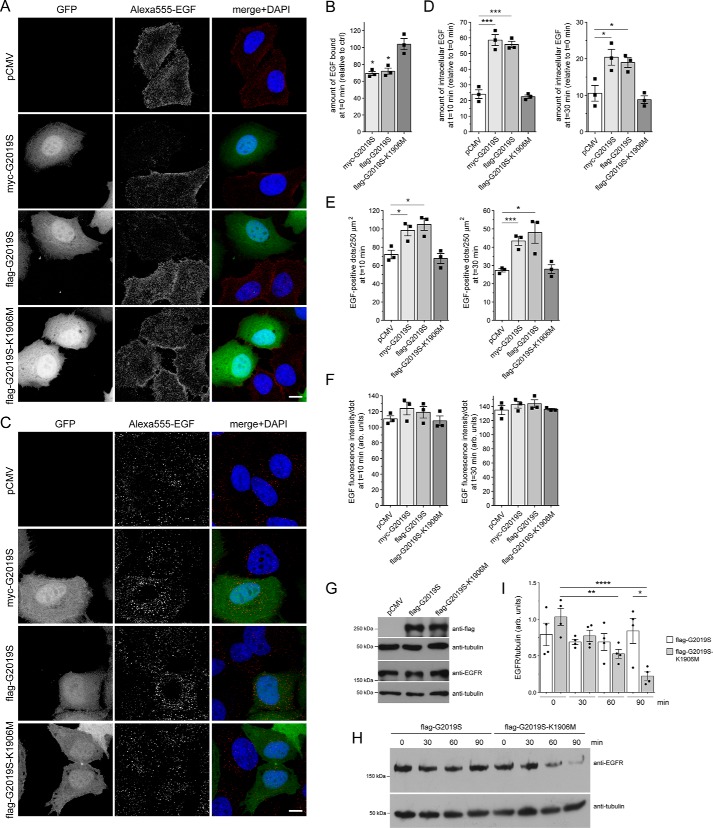
**Pathogenic G2019S LRRK2, but not a kinase-inactive G2019S-K1906M variant, causes a deficit in EGF binding and degradation.** *A*, HeLa cells were transfected with either pCMV or cotransfected with GFP and either myc-tagged G2019S LRRK2, FLAG-tagged G2019S, or G2019S-K1906M LRRK2 as indicated; incubated with Alexa555-EGF for 30 min at 4 °C; washed to remove unbound fluorescent EGF; and fixed and processed as described under “Materials and methods.” *Scale bar*, 10 μm. *B*, quantification of surface-bound fluorescent EGF (*t* = 0 min) of cells transfected with the various constructs as indicated and normalized to EGF surface binding of pCMV-transfected cells (*ctrl*). *n* = 3 independent experiments. *, *p* < 0.05. *C*, HeLa cells transfected with the indicated constructs were allowed to bind Alexa555-EGF at 4 °C, washed to remove unbound fluorescent EGF, and then shifted to 37 °C for 10 min to allow for the internalization and degradation of fluorescent EGF. *Scale bar*, 10 μm. *D*, quantification of Alexa555-EGF was performed after 10 (*left*) and 30 min (*right*) upon internalization and normalized to the amount of Alexa555-EGF binding for each condition at *t* = 0 min, thus reflecting the percentage of internalized bound fluorescent EGF. *n* = 3 independent experiments. *, *p* < 0.05; ***, *p* < 0.005. *E*, quantification of the total number of fluorescent EGF-positive puncta per 250 μm^2^ upon expression of distinct constructs as indicated after 10 (*left*) and 30 min (*right*) of internalization. *n* = 3 independent experiments. *, *p* < 0.05; ***, *p* < 0.005. *F*, because the immunofluorescence signal intensity directly correlates to the size of the individual structures, signal intensity per punctum was quantified at 10 (*left*) and 30 min (*right*) upon internalization, which revealed no change among the different conditions, further indicating a deficit in EGF degradation rather than an increase in the amount of internalized fluorescent EGF per cell. *G*, HEK293T cells were transfected with the indicated constructs followed by analysis of endogenous EGFR expression levels. *H*, HEK293T cells were transfected with either pathogenic G2019S LRRK2 or with kinase-inactive G2019S-K1906M variant and serum-starved for 1 h in the presence of cycloheximide to block novel protein synthesis, and EGFR internalization was stimulated with nonlabeled EGF for the indicated time points. Cell extracts were analyzed by Western blotting for EGFR levels, and tubulin was used as a loading control. *I*, quantification of EGFR degradation in HEK293T cells transfected with either G2019S LRRK2 or G2019S-K1906M, at distinct time points as indicated, and with values normalized to tubulin as a loading control. *n* = 4 independent experiments. *, *p* < 0.05; **, *p* < 0.01; ****, *p* < 0.001. All *error bars* represent S.E.M.

Upon binding of EGF, the EGFR is internalized by clathrin-mediated endocytosis and sorted to lysosomes for degradation in a manner dependent on RAB7 (32). EGFR surface availability can be assessed by binding of Alexa555-EGF to cells at 4 °C, and endocytic trafficking and degradation can be followed by quantification of endocytosed Alexa555-EGF over time. HeLa cells were cotransfected with GFP and with either myc-tagged or FLAG-tagged G2019S LRRK2 or with a FLAG-tagged kinase-inactive variant (G2019S-K1906M) of pathogenic LRRK2, respectively, followed by assessment of binding and degradation of fluorescently labeled EGF. Binding of Alexa555-EGF at 4 °C was reduced in the presence of either myc-tagged or FLAG-tagged G2019S LRRK2 but not a kinase-inactive version thereof, indicating that it was tag-independent and due to the LRRK2 kinase activity (Fig. 1*A* and *B*). Upon incubation of cells at 37 °C for either 10 or 30 min to monitor fluorescent EGF degradation, a pronounced delay in the clearance of Alexa555-EGF per cell was observed in the presence of G2019S but not kinase-inactive G2019S-K1906M LRRK2, respectively (Fig. 1, *C* and *D*). A similar pathogenic LRRK2-mediated deficit in EGF clearance was observed when quantifying intracellular fluorescent EGF-positive puncta per area with no change in the fluorescence intensity of the individual dots (Fig. 1, *E* and *F*). To further corroborate that the increase in intracellular fluorescent EGF was due to impaired EGFR degradation, biochemical EGFR degradation assays were performed in transfected HEK293T cells expressing G2019S or kinase-inactive G2019S-K1906M LRRK2, respectively. Pathogenic LRRK2 expression did not alter steady-state EGFR expression levels (Fig. 1*G*). However, and as described previously (24), cells expressing G2019S LRRK2 displayed a deficit in EGFR degradation as compared with cells expressing kinase-inactive G2019S-K1906M LRRK2 (Fig. 1, *H* and *I*). Therefore, pathogenic LRRK2 interferes with the endolysosomal trafficking and degradation of EGF and the EGFR.

**Figure 2.**
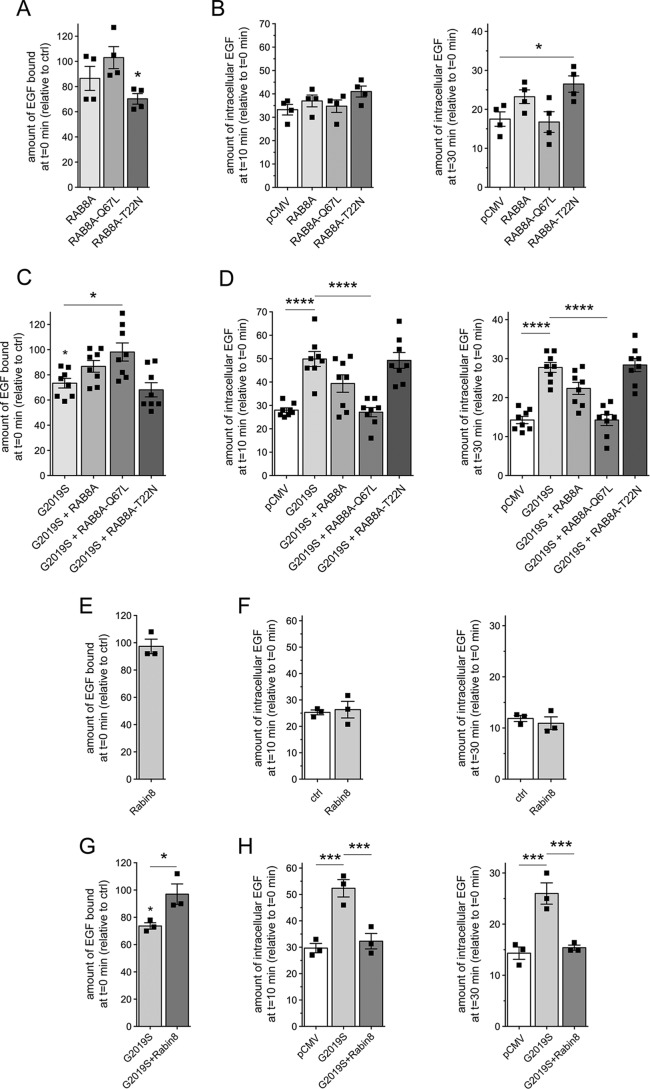
**Active RAB8A and Rabin8 rescue the LRRK2-mediated deficit in EGF binding and degradation.** *A*, HeLa cells were transfected with either empty pCMV vector (*ctrl*) or the indicated RAB8A constructs followed by quantification of the amount of surface-bound fluorescent EGF. *n* = 4 independent experiments. *, *p* < 0.05. *B*, cells were transfected as indicated followed by quantification of internalized Alexa555-EGF in transfected cells after 10 (*left*) and 30 min (*right*) of internalization. Values are normalized to the amount of Alexa555-EGF binding at *t* = 0. *n* = 4 independent experiments. *, *p* < 0.05. *C*, cells were cotransfected with G2019S LRRK2 and the indicated RAB8A constructs, and surface-bound fluorescent EGF was quantified. *n* = 8 independent experiments. *, *p* < 0.05. *D*, cells were cotransfected with G2019S LRRK2 and the indicated RAB8A constructs followed by quantification of internalized Alexa555-EGF after 10 (*left*) and 30 min (*right*) of internalization. *n* = 8 independent experiments. ****, *p* < 0.001. *E*, cells were transfected with either empty pCMV vector (*ctrl*) or with Rabin8, and surface-bound fluorescent EGF was quantified. *n* = 3 independent experiments. *F*, cells were transfected as indicated followed by quantification of internalized fluorescent EGF at 10 (*left*) and 30 min (*right*). *n* = 3 independent experiments. *G*, cells were transfected with either empty pCMV vector (*ctrl*) or cotransfected with G2019S pathogenic LRRK2 and either pCMV vector or Rabin8 as indicated, and surface-bound fluorescent EGF was quantified. *n* = 3 experiments. *, *p* < 0.05. *H*, cells were transfected as indicated followed by quantification of internalized fluorescent EGF as described above. *n* = 3 independent experiments. ***, *p* < 0.005. All *error bars* represent S.E.M.

We next wondered how these LRRK2-mediated endolysosomal trafficking deficits may be modulated by RAB8A. In HeLa cells, GFP-tagged WT RAB8A and GTP-locked, constitutively active RAB8A-Q67L were largely localized to a tubular endocytic recycling compartment partially overlapping with the transferrin receptor, with tubular localization more evident in live or in fixed but only briefly permeabilized cells (Fig. S2, A–C). In contrast, GDP-locked inactive RAB8A-T22N was cytosolic and not properly targeted to a tubular recycling compartment (Fig. S2B). When expressed on their own, neither RAB8A nor RAB8A-Q67L caused alterations in EGF binding or EGFR trafficking, whereas RAB8A-T22N caused a modest decrease in EGF surface binding and a slight delay in EGFR degradation, evident only at *t* = 30 min (Fig. 2, *A* and *B*). Importantly, the pathogenic G2019S LRRK2–mediated decrease in EGF binding and the delay in EGFR trafficking were fully rescued when expressing RAB8A-Q67L (Fig. 2, *C* and *D*). This was not observed with WT RAB8A or with RAB8A-T22N (Fig. 2, *C* and *D*), although both WT and RAB8A-Q67L were expressed to similar degrees (Fig. S2D), and none of the RAB8A variants interfered with the coexpression of G2019S LRRK2 (Fig. S3, A and B). In this cell system, and taking into account a roughly 50% transfection efficiency, overexpression of LRRK2 constructs was about 1–1.5-fold above endogenous levels, and overexpression of RAB8A was around 2.5–4-fold above endogenous levels, respectively (Fig. S2, *D* and *E*).

Rabin8 functions as a GEF for RAB8A and activates it by catalyzing GDP release for subsequent GTP loading (33). Conversely, Rabin8 is activated by RAB11, which controls vesicle exit from recycling endosomes (34–37). However, it remains unknown whether such cascade operates in all RAB8A-dependent membrane trafficking events. We therefore tested whether Rabin8 or RAB11 could rescue the LRRK2-mediated deficit in endolysosomal trafficking. Overexpressed Rabin8 was largely cytosolic (Fig. S2B) and had no effect on endolysosomal trafficking when expressed on its own (Fig. 2*E* and *F*). However, Rabin8 expression rescued the G2019S LRRK2–mediated deficits in EGF binding and degradation (Fig. 2, *G* and *H*) without interfering with the expression levels of G2019S LRRK2 (Fig. S3C), indicating that activating endogenous RAB8A by Rabin8 expression also rescues the LRRK2-mediated endolysosomal trafficking defects.

**Figure 3.**
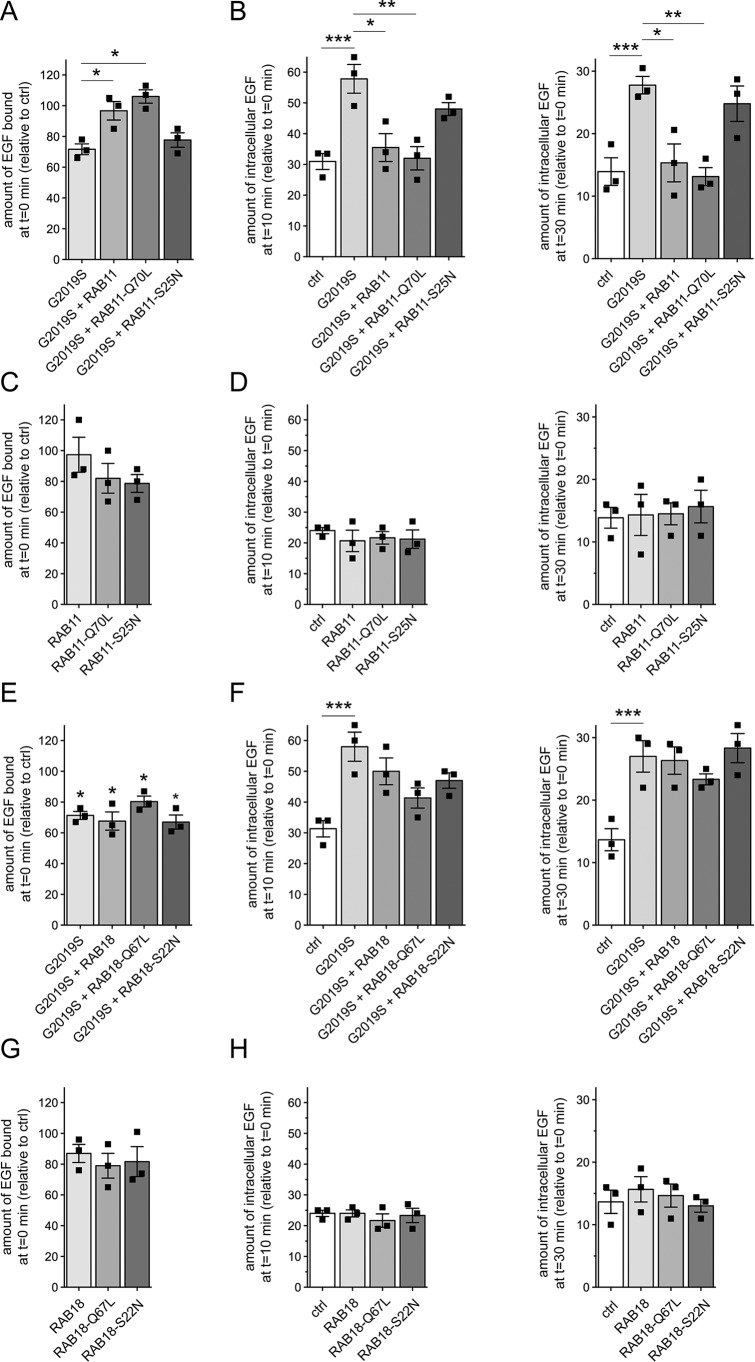
**RAB11 rescues the LRRK2-mediated delay in EGFR trafficking.** *A*, HeLa cells were cotransfected with G2019S LRRK2 and the indicated RAB11 constructs, and surface-bound fluorescent EGF was quantified. *n* = 3 independent experiments. *, *p* < 0.05. *B*, cells were transfected with either empty pCMV vector (*ctrl*) or cotransfected with G2019S LRRK2 and the indicated RAB11 constructs followed by quantification of internalized fluorescent EGF at 10 (*left*) and 30 min (*right*). *n* = 3 independent experiments. *, *p* < 0.05; **, *p* < 0.01; ***, *p* < 0.005. *C*, cells were transfected with either empty pCMV vector (*ctrl*) or the indicated RAB11 constructs, and the amount of surface-bound fluorescent EGF was quantified. *n* = 3 independent experiments. *D*, cells were transfected as indicated followed by quantification of internalized EGF at 10 (*left*) and 30 min (*right*). *n* = 3 independent experiments. *E*, cells were transfected with either empty pCMV vector (*ctrl*) or cotransfected with G2019S LRRK2 and the indicated RAB18 constructs, and surface-bound fluorescent EGF was quantified. *n* = 3 independent experiments. *, *p* < 0.05. *F*, cells were transfected as indicated followed by quantification of internalized fluorescent EGF at 10 (*left*) and 30 min (*right*). *n* = 3 independent experiments. ***, *p* < 0.005. *G*, same as in *E*, but cells were transfected with either empty pCMV vector (*ctrl*) or the indicated RAB18 constructs. *n* = 3 independent experiments. *H*, same as in *F*, but cells were transfected with either empty pCMV vector (*ctrl*) or the indicated RAB18 constructs. *n* = 3 independent experiments. All *error bars* represent S.E.M.

We next analyzed the effects of RAB11 on the LRRK2-mediated deficits in EGFR trafficking. GFP-tagged RAB11 colocalized with transferrin receptor (Fig. S2A). Both WT and GTP-locked active RAB11 (RAB11-Q70L) were membrane-associated, whereas GDP-locked inactive RAB11 (RAB11-S25N) was largely cytosolic (Fig. S2B). When coexpressed with pathogenic LRRK2, both WT and active, but not inactive RAB11, rescued the deficit in EGF binding and degradation (Fig. 3, *A* and *B*), but none of them had an effect on their own (Fig. 3, *C* and *D*) even though they were expressed to comparable degrees and did not alter the expression levels of G2019S LRRK2 (Fig. S3, D and E). As another means to show that the effects reported here were specific, we analyzed the role of RAB18, an endoplasmic reticulum–resident RAB protein (38). RAB18 was localized to a reticular pattern reminiscent of the endoplasmic reticulum as well as the nuclear envelope (Fig. S2, A and B) (38). Neither WT, GTP-locked (RAB18-Q67L), nor GDP-locked RAB18 (RAB18-S22N) could rescue the pathogenic LRRK2-mediated deficits (Fig. 3, *E* and *F*), and they also displayed no effect on their own (Fig. 3, *G* and *H*) even though they were expressed to similar degrees and did not affect the levels of coexpressed G2019S LRRK2 (Fig. S3, F and G). Altogether, these data indicate that the RAB11–Rabin8–RAB8A cascade specifically rescues the endolysosomal trafficking defects associated with pathogenic LRRK2.

### G2019S LRRK2–mediated endolysosomal trafficking deficits are mimicked by knockdown of RAB8A

**Figure 4.**
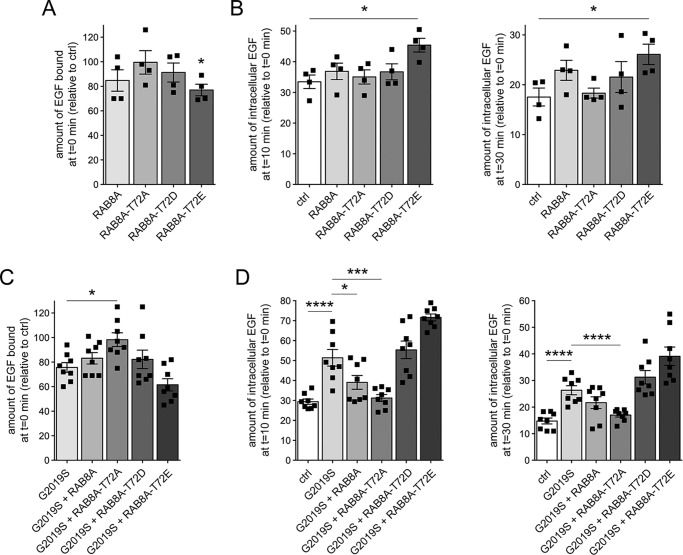
**Phosphodeficient RAB8A, but not WT or phosphomimetic RAB8A variants, revert the LRRK2-mediated effects on EGFR trafficking.** *A*, HeLa cells were transfected with either empty pCMV vector (*ctrl*) or the indicated RAB8A constructs, and surface-bound fluorescent EGF was quantified. *n* = 4 independent experiments. *, *p* < 0.05. *B*, cells were transfected with the indicated constructs followed by quantification of internalized fluorescent EGF. *n* = 4 independent experiments. *, *p* < 0.05. *C*, cells were cotransfected with G2019S LRRK2 and the indicated RAB8A constructs, and surface-bound fluorescent EGF was quantified. *n* = 8 independent experiments. *, *p* < 0.05. *D*, cells were transfected with the indicated constructs, and internalized fluorescent EGF was quantified at 10 (*left*) and 30 min (*right*). *n* = 8 independent experiments. *, *p* < 0.05; ***, *p* < 0.005; ****, *p* < 0.001. All *error bars* represent S.E.M.

We reasoned that if LRRK2-mediated phosphorylation of RAB8A causes its inactivation, a phosphomimetic RAB8A variant should be unable to rescue the delay in EGFR degradation. Thus, we expressed WT RAB8A, the phosphodeficient RAB8A-T72A variant, or the phosphomimetic RAB8A-T72D/RAB8A-T72E variants, respectively. Although WT RAB8A was largely localized to a tubular recycling compartment, the phosphomimetic variants displayed a prominently cytosolic localization, and the phosphodeficient RAB8A variant was also partially cytosolic (Fig. S2F). With the exception of RAB8A-T72E, none of these variants significantly altered EGF binding or EGFR degradation when expressed on their own (Fig. 4, *A* and *B*), with all constructs apart from RAB8A-T22N and RAB8A-T72E expressed to similar degrees (Fig. S2E). However, the phosphodeficient, but not the phosphomimetic, RAB8A versions fully rescued the effect of pathogenic LRRK2 (Fig. 4, *C* and *D*) without altering the coexpression levels of G2019S LRRK2 (Fig. S3, H and I).

**Figure 5.**
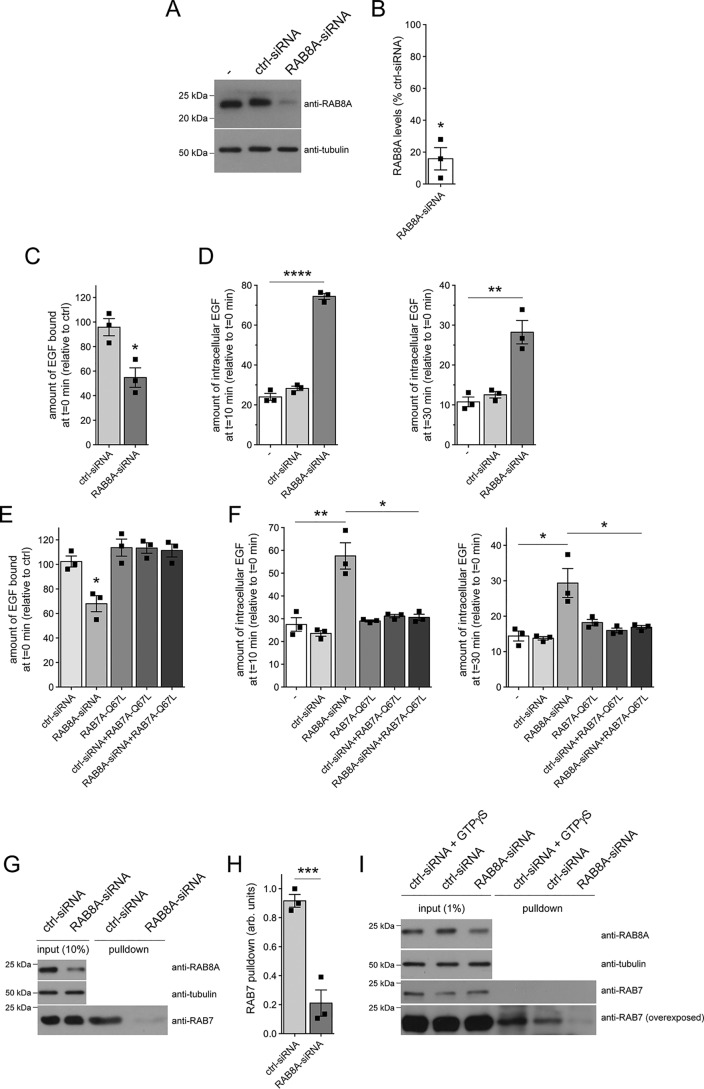
**Knockdown of RAB8A mimics the endolysosomal trafficking deficits mediated by G2019S LRRK2.** *A*, HeLa cells were either nontransfected (−) or transfected with ctrl-siRNA or RAB8A-siRNA, and cell extracts (30 μg) were analyzed by Western blotting for RAB8A protein levels and tubulin as a loading control. *B*, quantification of the type of experiments depicted in *A*. RAB8A levels in the presence of RAB8A-siRNA were normalized to levels in the presence of ctrl-siRNA. *n* = 3 independent experiments. *, *p* < 0.05. *C*, cells were either left untreated (−) or transfected with ctrl-siRNA or RAB8A-siRNA, and surface-bound fluorescent EGF was quantified. *n* = 3 independent experiments. *, *p* < 0.05. *D*, cells were either left untreated (−) or transfected with ctrl-siRNA or RAB8A-siRNA followed by quantification of internalized fluorescent EGF at 10 (*left*) and 30 min (*right*). *n* = 3 independent experiments. **, *p* < 0.01; ****, *p* < 0.001. *E*, cells were either left untreated or cotransfected with ctrl-siRNA or RAB8A-siRNA in the absence or presence of GFP-tagged active RAB7A (RAB7A-Q67L), and surface-bound fluorescent EGF was quantified. *n* = 3 independent experiments. *, *p* < 0.05. *F*, cells were either left untreated or cotransfected with ctrl-siRNA or RAB8A-siRNA in the absence or presence of RAB7A-Q67L, and internalized fluorescent EGF was quantified at 10 (*left*) and 30 min (*right*). *n* = 3 independent experiments. *, *p* < 0.05; **, *p* < 0.01. *G*, cells were either treated with ctrl-siRNA or RAB8A-siRNA as indicated, and the RAB7-binding domain of RILP coupled to GST was used to pull down the GTP-bound form of RAB7 from cell lysates (300 μg). Input (10%) was run alongside pulldowns to demonstrate equal levels of total RAB7 protein in ctrl-siRNA– or RAB8A-siRNA–treated cells, and the levels of RAB8A and tubulin were analyzed on a separate gel. *H*, experiments of the type depicted in *G* were quantified, and the amount of RAB7 isolated by GST-RILP was expressed relative to input. *n* = 3 independent experiments. ***, *p* < 0.005. *I*, cells were either treated with ctrl-siRNA or RAB8A-siRNA as indicated, and a conformation-specific antibody was used to immunoprecipitate active RAB7 from cell lysates (2 mg). As a positive control, ctrl-siRNA–treated cell extracts were incubated with 100 μm GTPγS to activate RAB7A before immunoprecipitation. Input (1%) was run alongside pulldowns to demonstrate equal levels of total RAB7 protein in ctrl-siRNA– or RAB8A-siRNA–treated cells, and the levels of RAB8A and tubulin were analyzed on a separate gel. All *error bars* represent S.E.M.

Various studies indicate that phosphodeficient and phosphomimetic RAB8A may not be able to properly mimic the dephosphorylated and phosphorylated status of RAB8A, respectively (25, 39–41). Therefore, and as another means to analyze the effect of LRRK2-mediated RAB8A inactivation on EGF binding and EGFR trafficking, we performed siRNA experiments. Specific siRNA of RAB8A caused a >80% decrease in RAB8A protein levels 48 h post-transfection (Fig. 5, *A* and *B*). Strikingly, RAB8A knockdown caused a pronounced deficit in EGF surface binding and EGFR degradation (Fig. 5, *C* and *D*). Moreover, the deficits induced upon siRNA of RAB8A could be rescued when overexpressing active, GTP-locked RAB7A (RAB7A-Q67L) (Fig. 5, *E* and *F*). Knockdown of RAB8A was not associated with a change in the steady-state levels of several other RAB proteins, including RAB7A (Fig. S4A), indicating that the deficits in endolysosomal trafficking are not due to off-target effects on RAB7A protein levels. In addition, an siRNA-resistant version of RAB8A (39), but not WT, siRNA-sensitive RAB8A, rescued the effect of RAB8A knockdown on EGF binding and EGFR trafficking (Fig. S5, A–D), further indicating that the effects were due to the specific knockdown of RAB8A.

To gain direct evidence for a change in RAB7A activity upon siRNA of RAB8A, we employed an effector pulldown assay using the RAB7-binding domain of RILP to selectively isolate RAB7-GTP from cell lysates (24, 42). Pulldown assays were performed from either nontreated cells or cells treated with control siRNA or with RAB8A-siRNA, respectively (Fig. 5, *G* and *H*). The fraction of endogenous RAB7 bound to GTP was drastically reduced upon RAB8A knockdown (Fig. 5, *G* and *H*), similar to what we described previously for G2019S LRRK2 (21). As an alternative means, active RAB7 was immunoprecipitated from cell extracts previously treated with control siRNA or RAB8A-siRNA using a conformation-specific antibody (43), which also showed a significant decrease of active RAB7 upon RAB8A knockdown (Fig. 5*I*). Therefore, loss of RAB8A function effectively phenocopies the pathogenic G2019S LRRK2–mediated defects in endolysosomal trafficking by decreasing RAB7 activity (21).

### G2019S LRRK2 or RAB8A knockdown causes accumulation of EGF in a RAB4 compartment

We next wondered whether the delay in EGF degradation in the presence of pathogenic LRRK2 may reflect a redistribution of EGF into a nonendolysosomal vesicular compartment, thereby precluding its efficient degradation. As expected, the active version of endolysosomal RAB7A extensively colocalized with Alexa647-EGF 20 min upon internalization (Fig. S4, B–D). In contrast, colocalization of fluorescent EGF with RAB8A or RAB11 was minor and was not altered in the presence of G2019S LRRK2 (Fig. S4, B–D), suggesting that pathogenic LRRK2 did not cause the accumulation of EGF in RAB8A- or RAB11-positive endocytic recycling compartments, respectively.

**Figure 6.**
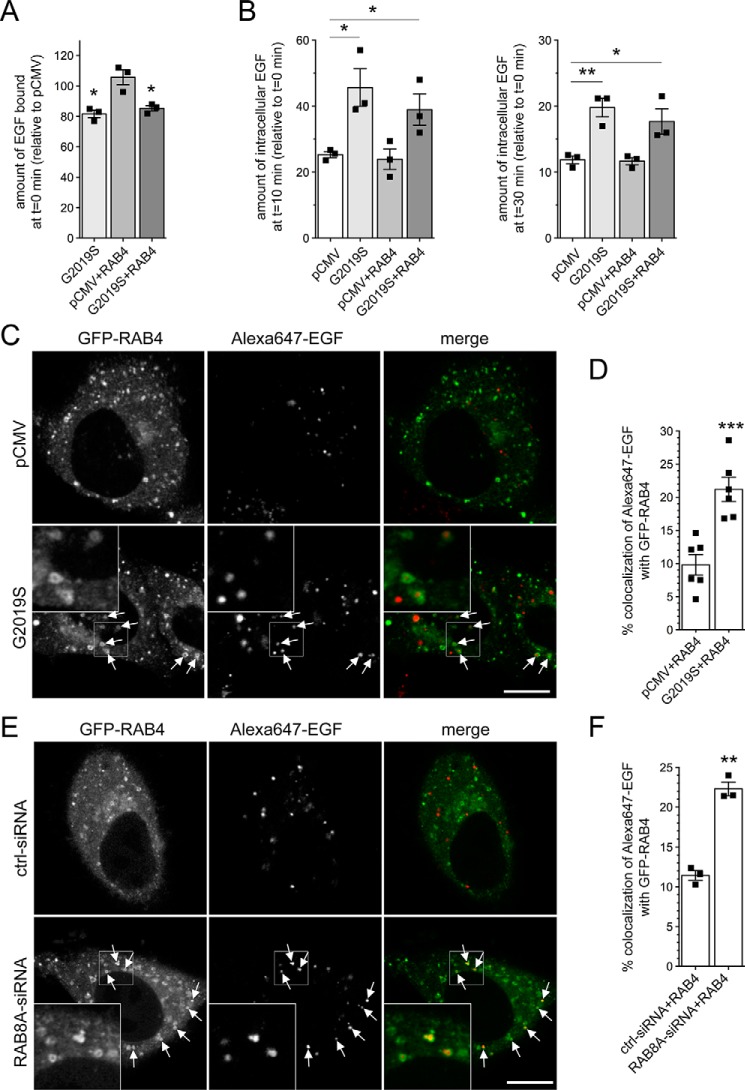
**Pathogenic LRRK2 or knockdown of RAB8A causes accumulation of EGF in a RAB4-positive endocytic compartment.** *A*, HeLa cells were transfected with either empty pCMV vector or pathogenic LRRK2 or cotransfected with GFP-tagged RAB4, and surface-bound fluorescent EGF was quantified. *n* = 3 independent experiments. *, *p* < 0.05. *B*, cells were transfected as indicated followed by quantification of internalized fluorescent EGF at 10 (*left*) and 30 min (*right*). *n* = 3 independent experiments. *, *p* < 0.05; **, *p* < 0.01. *C*, example of HeLa cells cotransfected with GFP-RAB4 and either empty pCMV vector or pathogenic LRRK2. Live pictures were taken 20 min upon fluorescent EGF internalization, and *arrows* point to GFP-RAB4–positive vesicles containing Alexa647-EGF. *Scale bar*, 10 μm. *D*, quantification of colocalization of Alexa647-EGF with GFP-RAB4 (Manders' coefficient 1 × 100) from 15–20 cells per experiment. *n* = 6 independent experiments. ***, *p* < 0.005. *E*, example of HeLa cells cotransfected with GFP-RAB4 and either ctrl-siRNA or RAB8A-siRNA. Live pictures were taken as described above. *Arrows* point to GFP-RAB4–positive vesicles containing Alexa647-EGF. *Scale bar*, 10 μm. *F*, quantification of colocalization of Alexa647-EGF with GFP-RAB4 (Manders' coefficient 1 × 100) from 15–20 cells per experiment. *n* = 3 independent experiments. **, *p* < 0.01. All *error bars* represent S.E.M.

Cross-talk between both the recycling and degradative endosomal trafficking pathways has been described to converge on an endocytic RAB4-positive compartment (31). We therefore next analyzed possible changes in the colocalization of EGF with GFP-tagged RAB4. Expression of RAB4 did not affect EGF binding or EGFR trafficking in either the absence or presence of G2019S LRRK2 (Fig. 6, *A* and *B*) and did not alter G2019S LRRK2 expression levels (Fig. S3J). However, when quantifying the colocalization of Alexa647-EGF with GFP-tagged RAB4 in live mock-transfected *versus* pathogenic LRRK2-transfected cells, a significant increase was observed in G2019S LRRK2–expressing cells (Fig. 6, *C* and *D*). Similarly, there was a significant increase in the accumulation of fluorescent EGF in a RAB4-positive compartment upon siRNA of RAB8A as compared with control siRNA (Fig. 6, *E* and *F*). These data indicate that either G2019S LRRK2 expression or RAB8A inactivation causes alterations in endolysosomal trafficking events, culminating in the accumulation of EGF in a nondegradative, RAB4-positive recycling compartment.

### G2019S LRRK2 causes a deficit in EGFR recycling

**Figure 7.**
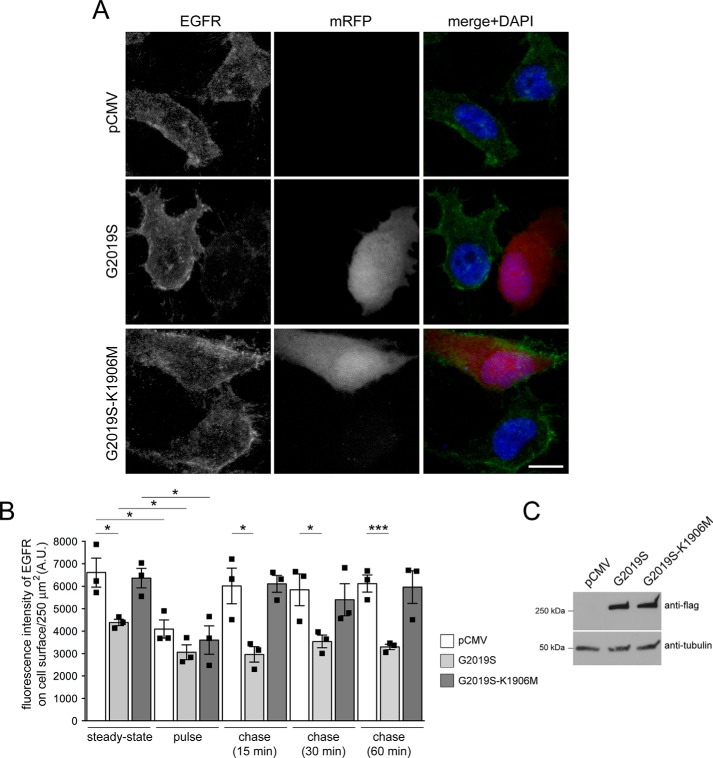
**Pathogenic G2019S LRRK2 causes a deficit in EGFR recycling.** *A*, example of HeLa cells transfected with pCMV or cotransfected with mRFP and either G2019S or kinase-inactive G2019S-K1906M LRRK2 and stained with an antibody against the extracellular domain of the EGFR in the absence of permeabilization to visualize only surface EGFR. *Scale bar*, 10 μm. *B*, quantification of fluorescence intensity of surface levels of EGFR at *t* = 0 min (*steady-state*), upon triggering internalization of the EGFR (*pulse*), or upon chase for various time points to assess recycling rates (*chase*) as described under “Materials and methods.” *n* = 3 independent experiments. *, *p* < 0.05; ***, *p* < 0.005. *C*, HeLa cells were transfected as indicated, and cell extracts (30 μg) were analyzed by Western blotting for FLAG-tagged LRRK2 levels and tubulin as a loading control. *A.U.*, arbitrary units. All *error bars* represent S.E.M.

The observed accumulation of fluorescent EGF in a RAB4-positive compartment suggests that pathogenic LRRK2 may cause additional alterations in endocytic recycling events. Because the concentration of EGF ligand is known to influence the balance between lysosomal degradation and recycling of the receptor, we next used low ligand concentrations to favor receptor recycling together with an antibody against the extracellular domain of the EGFR in the absence of permeabilization to visualize only surface EGFR (44). Cells were cotransfected with mRFP and either pathogenic G2019S LRRK2 or kinase-inactive G2019S-K1906M, serum-starved, and incubated on ice with 20 ng/ml nonlabeled EGF for 20 min in the presence of cycloheximide to prevent novel protein synthesis (steady state). Cells were then shifted to 37 °C, which allows them to internalize EGFR (pulse), followed by a chase for various time points to assess EGFR recycling rates back to the cell surface (chase). As observed for fluorescent EGF surface binding, the antibody against the extracellular domain of the EGFR revealed a decrease in EGFR surface levels under steady-state conditions in cells expressing G2019S LRRK2 but not kinase-inactive G2019S-K1906M, respectively (Fig. 7, *A* and *B*), even though both were expressed to similar degrees (Fig. 7*C*). Shifting cells to 37 °C caused receptor internalization in all cases, but after a 15-min chase, cells showed differential EGFR recycling rates back to the cell surface with an impairment in the presence of G2019S LRRK2 but not the kinase-inactive G2019S-K1906M variant (Fig. 7*B*). Quantification of the fluorescence intensity of EGFR on the cell surface showed that the receptor did not recycle to the cell surface even upon prolonged chase times (Fig. 7*B*). These data indicate that pathogenic LRRK2 causes not only a deficit in endolysosomal trafficking but also a defect in endocytic EGFR recycling.

### Mistargeting of EGF into a RAB4 compartment and endocytic recycling deficits mediated by either G2019S LRRK2 or RAB8A knockdown are rescued upon active RAB7A expression

**Figure 8.**
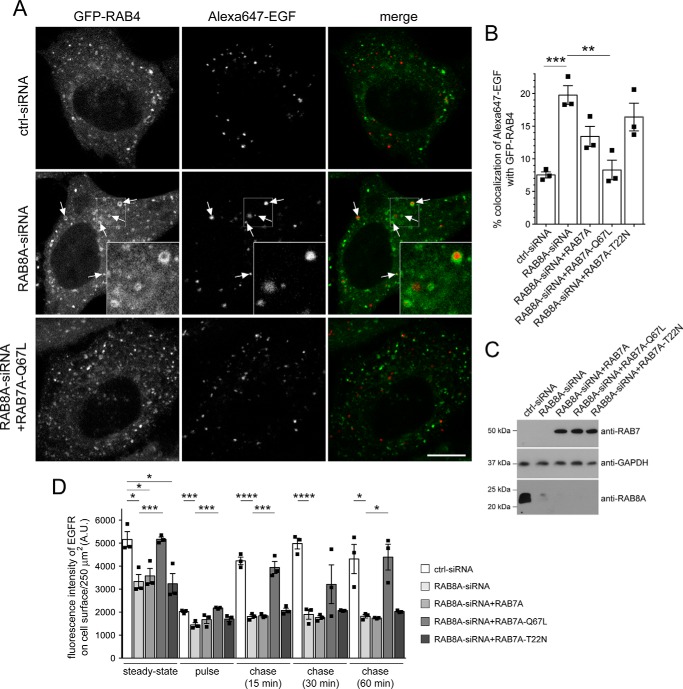
**Accumulation of EGF in a RAB4-positive endocytic compartment and deficits in EGFR recycling due to knockdown of RAB8A are rescued by active RAB7A expression.** *A*, example of HeLa cells cotransfected with GFP-RAB4 and either ctrl-siRNA or RAB8A-siRNA with or without RAB7A-Q67L expression as indicated. Live pictures were taken 20 min upon fluorescent EGF internalization, and *arrows* point to GFP-RAB4–positive vesicles containing Alexa647-EGF. An independent picture (543 HeNe laser line) was acquired to confirm coexpression of the distinct mRFP-tagged RAB7A constructs in all cases. *Scale bar*, 10 μm. *B*, quantification of colocalization of Alexa647-EGF with GFP-RAB4 and either ctrl-siRNA or RAB8A-siRNA in the presence or absence of distinct RAB7A constructs as indicated (Manders' coefficient 1 × 100) from 15–20 cells per experiment. *n* = 3 independent experiments. **, *p* < 0.01; ***, *p* < 0.005. *C*, HeLa cells were treated with ctrl-siRNA or RAB8A-siRNA as indicated and transfected with the indicated RAB7A constructs, and cell extracts (30 μg) were analyzed by Western blotting for RAB8A protein levels, mRFP-RAB7A protein levels (anti-RAB7 antibody), and GAPDH as a loading control. *D*, HeLa cells were treated with either ctrl-siRNA or RAB8A-siRNA as indicated with or without cotransfection with the indicated RAB7A constructs. EGFR recycling assays were performed as described under “Materials and methods,” revealing a deficit in EGFR surface levels and EGFR recycling upon RAB8A-siRNA, which was rescued upon expression of active RAB7A. *n* = 3 independent experiments. *, *p* < 0.05; ***, *p* < 0.005; ****, *p* < 0.001. *A.U.*, arbitrary units. All *error bars* represent S.E.M.

**Figure 9.**
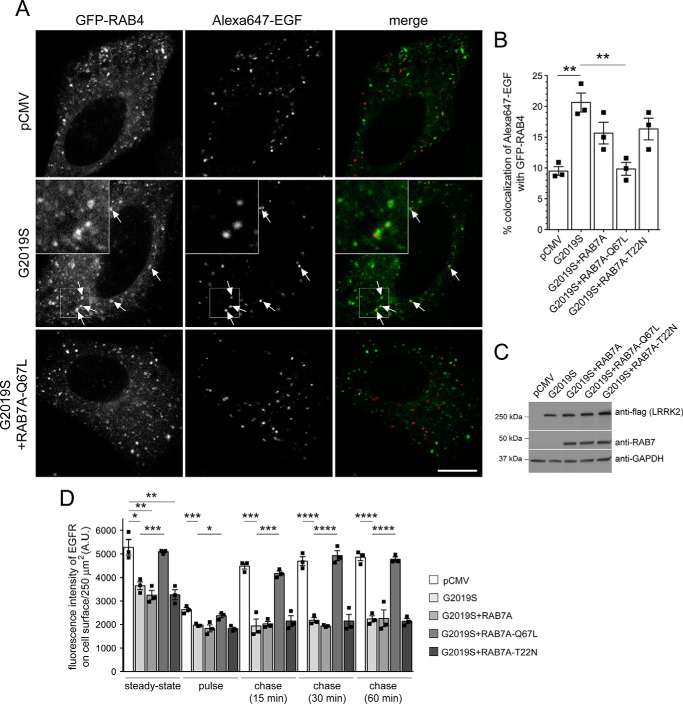
**Accumulation of EGF in a RAB4-positive endocytic compartment and deficits in EGFR recycling due to G2019S LRRK2 expression are rescued by active RAB7A expression.** *A*, example of HeLa cells cotransfected with GFP-RAB4 and either empty pCMV vector or pathogenic LRRK2 with or without RAB7A-Q67L expression as indicated. Live pictures were taken 20 min upon fluorescent EGF internalization, and *arrows* point to GFP-RAB4–positive vesicles containing Alexa647-EGF. An independent picture (543 HeNe laser line) was acquired to confirm coexpression of the distinct mRFP-tagged RAB7A constructs in all cases. *Scale bar*, 10 μm. *B*, quantification of colocalization of Alexa647-EGF with GFP-RAB4 in cells coexpressing empty pCMV vector or G2019S LRRK2 in the presence or absence of mRFP-tagged RAB7A constructs as indicated (Manders' coefficient 1 × 100) from 15–20 cells per experiment. *n* = 3 independent experiments. **, *p* < 0.01. *C*, HeLa cells were transfected with the indicated constructs, and cell extracts (30 μg) were analyzed by Western blotting for FLAG-tagged G2019S-LRRK2, mRFP-RAB7A protein levels (anti-RAB7 antibody), and GAPDH as a loading control. *D*, HeLa cells were transfected with either empty pCMV vector or pathogenic G2019S LRRK2 in the presence or absence of mRFP-tagged RAB7A constructs as indicated, and EGFR surface levels and EGFR recycling were determined at the indicated time points. *n* = 3 independent experiments. *, *p* < 0.05; **, *p* < 0.01; ***, *p* < 0.005; ****, *p* < 0.001. *A.U.*, arbitrary units. All *error bars* represent S.E.M.

Because expression of active RAB7A rescued the endolysosomal EGFR trafficking deficits observed upon either siRNA of RAB8A or G2019S LRRK2 expression (21), we wondered whether active RAB7A expression would also lead to a reversal in the accumulation of fluorescent EGF in a RAB4-positive compartment and a rescue of the endocytic EGFR recycling deficit. Quantification of the colocalization of Alexa647-EGF with GFP-tagged RAB4 upon siRNA of RAB8A was rescued when coexpressing active RAB7A-Q67L but not WT or inactive RAB7A-T22N versions, respectively, even though all constructs were expressed to similar degrees (Fig. 8, *A–C*). Furthermore, siRNA of RAB8A caused a decrease in EGFR surface levels and endocytic recycling deficits of the EGFR, which were rescued upon coexpression of active RAB7A-Q67L but not WT or inactive RAB7A-T22N versions, respectively (Fig. 8*D*). Similarly, expression of active, but not WT or inactive RAB7A, caused a reversal in the accumulation of fluorescent EGF in a RAB4-positive compartment upon pathogenic G2019S LRRK2 expression with constructs expressed to similar degrees (Fig. 9, *A–C*). In addition, the LRRK2-mediated decrease in EGFR surface levels and the endocytic recycling deficits of the EGFR were rescued by active but not WT or inactive RAB7A versions, respectively (Fig. 9*D*). Thus, pathogenic LRRK2 expression causes a defect in endolysosomal degradation, which is accompanied by mistargeting of EGF into a RAB4 compartment and by a deficit in endocytic recycling, all phenocopied by knockdown of RAB8A and rescued in the presence of active RAB7A.

### Dominant-negative RAB7A causes accumulation of EGF in a RAB4 compartment and endocytic recycling deficits, which are rescued by active RAB8A

**Figure 10.**
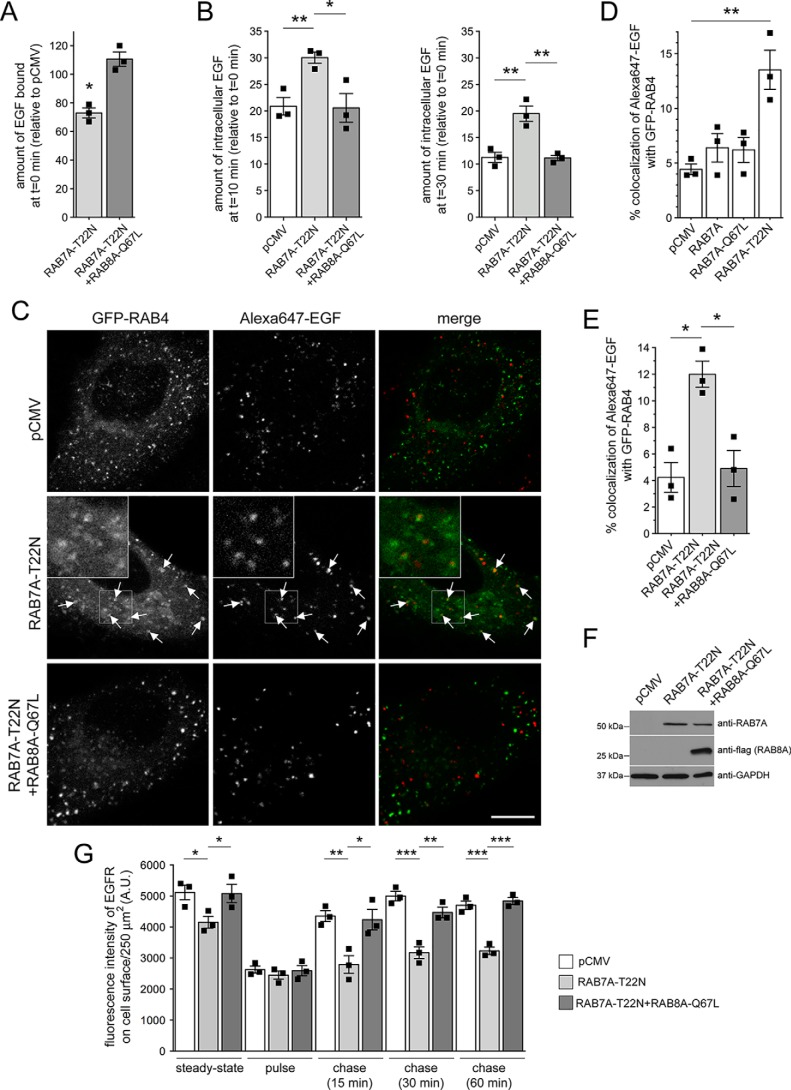
**Expression of dominant-negative RAB7A causes defects in EGFR trafficking, accumulation of EGF in a RAB4-positive endocytic compartment, and deficits in EGFR recycling, which are reversed upon active RAB8A expression.** *A*, HeLa cells were transfected with either empty pCMV vector (*ctrl*) or with dominant-negative RAB7A (RAB7A-T22N) in the presence or absence of active RAB8A (RAB8A-Q67L), and surface-bound fluorescent EGF was quantified. *n* = 3 independent experiments. *, *p* < 0.05. *B*, cells were transfected with the indicated constructs followed by quantification of internalized fluorescent EGF at 10 (*left*) and 30 min (*right*). *n* = 3 independent experiments. *, *p* < 0.05; **, *p* < 0.01. *C*, example of HeLa cells cotransfected with GFP-RAB4 and either mRFP-RAB7A-T22N or mRFP-RAB7A-T22N and FLAG-tagged RAB8A-Q67L as indicated. Live pictures were taken 20 min upon fluorescent EGF internalization, and *arrows* point to GFP-RAB4–positive vesicles containing Alexa647-EGF. An independent picture (543 HeNe laser line) was acquired to confirm coexpression of the mRFP-tagged RAB7A constructs in all cases. *Scale bar*, 10 μm. *D*, quantification of colocalization of Alexa647-EGF with GFP-RAB4 in the presence or absence of the distinct RAB7A constructs as indicated (Manders' coefficient 1 × 100) from 15–20 cells per experiment. *n* = 3 independent experiments. **, *p* < 0.01. *E*, quantification of colocalization of Alexa647-EGF with GFP-RAB4 in the presence or absence of RAB7A-T22N and RAB8A-Q67L constructs as indicated (Manders' coefficient 1 × 100) from 15–20 cells per experiment. *n* = 3 independent experiments. *, *p* < 0.05. *F*, HeLa cells were transfected with the indicated constructs, and cell extracts (30 μg) were analyzed by Western blotting for mRFP-tagged RAB7A-T22N, FLAG-tagged RAB8A-Q67L, and GAPDH as a loading control. *G*, HeLa cells were transfected with either empty pCMV vector or dominant-negative RAB7A-T22N in the absence or presence of RAB8A-Q67L, and EGFR surface levels and EGFR recycling were determined at the indicated time points. *n* = 3 independent experiments. *, *p* < 0.05; **, *p* < 0.01; ***, *p* < 0.005. *A.U.*, arbitrary units. All *error bars* represent S.E.M.

Finally, we wondered whether decreasing active RAB7A levels *per se* may account for the mistargeting of the EGFR into a RAB4A compartment and endocytic recycling deficits as observed upon pathogenic LRRK2 expression or knockdown of RAB8A. Expression of dominant-negative RAB7A (RAB7A-T22N) (45) interfered with EGF surface binding and EGFR degradation, which were rescued upon overexpression of active RAB8A (Fig. 10, *A* and *B*). Furthermore, expression of dominant-negative RAB7A, but not WT or catalytically active RAB7A (RAB7A-Q67L), caused accumulation of EGF in a RAB4 compartment (Fig. 10, *C* and *D*), which was rescued upon overexpression of active RAB8A (Fig. 10, *E* and *F*). Similarly, dominant-negative RAB7A caused a decrease in EGFR surface levels and EGFR recycling, which were rescued upon overexpression of active RAB8A (Fig. 10*G*). Thus, decreasing RAB7A activity causes the same trafficking deficits as observed upon pathogenic LRRK2 expression or knockdown of RAB8A, and such effects can be rescued by either active RAB7A or RAB8A expression, respectively. Altogether, our data indicate that G2019S LRRK2 expression causes inactivation of RAB8A associated with inactivation of RAB7A and followed by alterations in endolysosomal trafficking events culminating in the accumulation of EGF in a nondegradative, RAB4-positive recycling compartment as well as impaired endocytic recycling.

## Discussion

In the present study, we reveal a link between RAB8A inactivation and endolysosomal deficits that may form the basis for how pathogenic LRRK2 deregulates degradative membrane trafficking pathways. We show that RAB8A serves as a prominent LRRK2 kinase substrate *in vitro*, in contrast to other RAB proteins involved in regulating endocytic recycling (RAB11) or endolysosomal trafficking events (RAB7A). We corroborate Thr-72 as the major LRRK2 phosphorylation site within RAB8A. This residue lies within the switch II domain, a highly conserved region of RAB proteins that regulates the interaction with multiple proteins (26, 46). Phosphorylation at this site has been shown to impair interaction with GDI and Rabin8 *in vitro* and may thus lead to RAB8A inactivation *in vivo* (25, 40).

Expression of both active or phosphodeficient RAB8A and the RAB8A activators Rabin8/RAB11 rescued the pathogenic LRRK2-mediated deficits in endolysosomal trafficking, suggesting that pathogenic LRRK2 may cause an inactivation of RAB8A *in vivo*. The phosphomimetic RAB8A variants were unable to rescue the LRRK2-mediated deficits and were largely localized to the cytoplasm rather than to a membranous tubular endocytic recycling compartment. Similar observations have been described previously for RAB7A (47). RAB7A was shown to be phosphorylated by an unknown protein kinase on an equivalent residue (Ser-72) in the switch II region, and a phosphomimetic RAB7A variant displayed impaired interaction with GDI and the RAB7A GEF and was also found localized to the cytoplasm (47). It is currently unclear whether LRRK2 preferentially phosphorylates RAB8A when in its GDP-bound, cytosolic state or when in its GTP-bound, membrane-associated state. In the former case and together with the observed impairment of GDI interaction, this would preclude phosphorylated RAB8A from being membrane-associated and result in the accumulation of inactive RAB8A in the cytosol. In the latter case, it may preclude the phosphorylated RAB8A version from being membrane-extracted and, together with the observed impairment of Rabin8 interaction, result in the accumulation of inactive RAB8A in the membrane. In either case, both scenarios would lead to RAB8A inactivation and concomitant downstream defects in membrane trafficking.

As an alternative means to test for the role of pathogenic LRRK2 in regulating RAB8A activity, we performed knockdown studies. Specific knockdown of RAB8A mimicked the LRRK2-induced deficits in endolysosomal trafficking, further strengthening the conclusion that pathogenic LRRK2 causes a loss-of-function phenotype of RAB8A in the context of altered membrane trafficking events. Recent studies have also reported that pathogenic LRRK2 causes a kinase-dependent and RAB-mediated deficit in protein transport and/or signaling defects at cilia (40, 41), and aberrant accumulation of phosphorylated RAB8A in a pericentrosomal/centrosomal compartment has been found to cause centrosome-related deficits (39). Both cilia and centrosomes are microtubule-derived structures (48), and the centrosome forms the primary microtubule-organizing center in many cells. Because endolysosomal transport processes are microtubule-dependent, phospho-RAB8A–mediated alterations in proper centrosome functioning may contribute to the trafficking deficits described here.

However, our data are consistent with a model whereby the LRRK2-mediated deficits in endolysosomal trafficking occur via RAB8A inactivation and concomitant alterations in RAB7A activity. As described previously for G2019S LRRK2 (21), the deficits in EGFR trafficking mediated by RAB8A knockdown were rescued when overexpressing active RAB7A. In addition, knockdown of RAB8A was associated with a decrease in RAB7A activity. The mistargeting of EGF into a RAB4-positive compartment and the deficits in EGFR recycling by either G2019S LRRK2 expression or knockdown of RAB8A were rescued upon active RAB7A expression. Conversely, expression of dominant-negative RAB7A caused identical deficits in endolysosomal EGF trafficking associated with the accumulation of EGF in a RAB4-positive compartment and deficits in EGFR recycling. These data are consistent with the idea that pathogenic LRRK2 causes a phosphorylation-mediated loss of proper RAB8A functioning and a concomitant decrease in RAB7A activity, the latter of which contributes to the observed trafficking deficits.

How a decrease in RAB8A activity and/or levels upon siRNA may trigger impaired RAB7A functioning remains unclear. A variety of scenarios are possible, including competition for shared GEFs, even though the currently identified GEFs for RAB8A and RAB7A are distinct (33, 49–53). Alternatively, the two RAB proteins may compete for GAPs, which display a certain promiscuity, with little correlation between their RAB binding and RAB–GAP activity (54, 55). In this context, it is interesting to note that TBC1D15, a GAP for RAB7A (42, 56), was found to preferentially interact with phosphodeficient as compared with phosphomimetic RAB8A (25). Although further work will be required to address this possibility, it is tempting to speculate that LRRK2-mediated phosphorylation of RAB8A may cause a decrease in its interaction with TBC1D15, thereby allowing it to act upon RAB7A, analogous to the recently reported retromer-mediated regulation of RAB7A activity via TBC1D5 (57).

The finding that deficits in RAB8A, a RAB protein that functions at the endocytic recycling compartment, cause alterations in late endocytic degradative trafficking seems puzzling at first. However, several reports have revealed cross-talk between the recycling and degradative trafficking pathways involving a RAB4-positive compartment. For example, overexpression of dominant-negative RAB4 variants was found to alter both endocytic recycling and degradation (58). Similarly, a GAP for RAB4 regulates both transferrin receptor recycling and degradative EGFR trafficking (59). Finally, abolishing the recruitment of a Rho-specific GAP protein to RAB8A-positive tubules interferes with transferrin receptor transport and EGFR degradation, with EGF accumulating in a RAB4 compartment (31). These as well as our data indicate the presence of a dynamic and intricate cross-talk between recycling and degradative membrane trafficking pathways that converge onto a RAB4-positive recycling compartment. Previous studies in various nonneuronal as well as neuronal cell types have shown that pathogenic LRRK2 causes endolysosomal defects, even though most of these studies employed endolysosomal morphological alterations as a readout for the pathogenic LRRK2-mediated deficits (60–65). In contrast, our studies, by probing alterations in the trafficking of the EGFR in a nonneuronal cell line, have allowed us to precisely dissect the dynamic alterations in the trafficking steps altered by pathogenic LRRK2 and point toward a role for impaired RAB8A functioning in those processes.

Various studies highlight an important role for RAB8A in PD. For example, the phosphorylation status of RAB8A is not only modulated by LRRK2 but also by the PD-related kinase PINK1 (66). Overexpression of RAB8A rescues α-synuclein–induced neuronal toxicity (67), and depletion of TMEM230, another PD-linked gene product, causes a decrease in RAB8A levels associated with deficits in retromer-mediated trafficking and Golgi-derived vesicle secretion (30). Thus, all currently available data indicate that reduced RAB8A protein levels and/or altered RAB8A phosphorylation/activation correlate with various cellular alterations related to PD pathogenesis, and our study demonstrates for the first time a novel role for a pathogenic LRRK2-mediated inactivation of RAB8A that impairs endolysosomal trafficking and correlates with a decrease in RAB7A activity. RAB8A and/or RAB7A activation may thus serve as alternative therapeutic drug strategies for LRRK2-related PD.

## Materials and methods

### DNA constructs and site-directed mutagenesis

Double myc–tagged WT or pathogenic LRRK2 constructs have been described previously (24), and triple FLAG–tagged LRRK2 constructs have been described previously as well (68). DNA was prepared from bacterial cultures grown at 28 °C (at 37 °C for RAB constructs) using a Midiprep kit (Promega) according to the manufacturer's instructions. GFP-RAB7A, GFP-RAB7A-Q67L, and GFP-RAB7A-T22N constructs have been described previously (24). Human GFP-RAB18, GFP-RAB18-Q67L, and GFP-RAB18-S22N were a generous gift from J. Presley (McGill University, Montreal, Canada). Human GFP-RAB11 and GFP-RAB11-S25N were gifts from Richard Pagano (Addgene plasmids 12674 and 12678) (69), and GFP-RAB11-Q70L was generated by site-directed mutagenesis (QuikChange, Stratagene). Human GFP-RAB8A, GFP-RAB8A-Q67L, and GFP-RAB8A-T22N were gifts from Maxence Nachury (Addgene plasmids 24898, 24899, and 24900) (34), and GFP-RAB8A-T72A, GFP-RAB8A-T72D, and GFP-RAB8A-T72E were generated by site-directed mutagenesis (QuikChange, Stratagene). An siRNA-resistant form of RAB8A (39) was generated by introducing three silent mutations into the target sequence of the seed region of the RAB8A-siRNA (Ambion, Thermo Fisher, ID s8679, catalog number 4390824). Specifically, the original sequence 5′-GCAAGAGAATTAAACTGCA-3′ was mutated to 5′-GCAAGAGAATTAAGTTACA-3′. Human GFP-Rabin8 was a gift from Keith Mostov (Addgene plasmid 26726) (36), and human GFP-RAB4 was a gift from Marci Scidmore (Addgene plasmid 49434) (70). Human GFP-RAB9 was a gift from Richard Pagano (Addgene plasmid 12663) (69). For generation of the distinct FLAG-tagged RAB proteins for *in vitro* phosphorylation studies, the respective RAB inserts were PCR-amplified with flanking EcoRI and BamHI restriction enzyme sites at the 5′ and 3′ ends, respectively, and the resultant PCR products were used to subclone into the EcoRI/BamHI sites of a 3XFLAG vector (Sigma-Aldrich). All 3XFLAG-tagged RAB8A constructs were generated by Gibson Assembly Master Mix (New England Biolabs). In all cases, the identity of constructs was verified by sequencing the entire coding region.

### Cell culture and transfection

HeLa cells were cultured and transfected as described previously (24). Briefly, cells were cultured in 100-mm dishes in full medium (DMEM containing 10% fetal bovine serum, nonessential amino acids, and high glucose) at 37 °C in 5% CO_2_. Confluent cells were harvested using 0.05% trypsin and 0.02 mm EDTA in PBS and subcultured at a ratio of 1:4–1:6. Cells were plated onto 6-well plates and the following day, at 70–80% confluence, transfected using Lipofectamine 2000 (Invitrogen) according to the manufacturer's specifications for 4 h in DMEM followed by replacement with fresh full medium. Double transfections were performed using 4 μg of LRRK2 plasmids and 1 μg of plasmids of interest. Transfected cells were replated the next day at a 1:2 ratio onto coverslips in 24-well plates. Proteins were expressed for 48 h before analysis as described below.

### Knockdown of RAB8A by RNAi

HeLa cells were seeded in 6-well plates at 30–40% confluence 1 day prior to transfection such that they were 70–80% confluent the following day. They were transfected with 25 nm siRNA and 1 μg of GFP-RAB7-Q67L or GFP-RAB4 as indicated using 4 μl of jetPRIME transfection reagent (Polyplus-Transfection SA, catalog number 114-15) in 200 μl of jetPRIME buffer. The mixture was incubated for 15 min at room temperature and added to 2 ml of full medium per well of a 6-well plate. For knockdown experiments in the presence of WT or siRNA-resistant mRFP-RAB8A variants, cells were transfected with a 50 nm concentration of the indicated siRNA using 4 μl of jetPRIME transfection reagent. Four hours later, medium were replaced with fresh DMEM, and cells were transfected with 1 μg of the indicated RAB8A constructs using Lipofectamine 2000 according to the manufacturer's instructions followed by medium replacement 4 h later. In all cases, cells were passaged 24 h later and processed for Western blotting analysis or fluorescent EGF binding and internalization assays 48 h after transfection. RNAi reagents included Silencer Select Negative Control Number 1 siRNA (Ambion, Thermo Fisher, catalog number 4390843) and Silencer Select RAB8A (Ambion, Thermo Fisher, ID s8679, catalog number 4390824). The latter was validated using TaqMan gene expression analysis and found to result in around a 95% reduction of mRNA levels 48 h post-transfection (Thermo Fisher). Knockdown efficacy of this siRNA reagent was confirmed by Western blotting with a sheep polyclonal RAB8A antibody that specifically detects RAB8A (antibody S969D, MRC-PPU Reagents).

### Immunofluorescence and laser confocal imaging

HeLa cells were fixed using 4% paraformaldehyde (PFA) in PBS for 20 min at room temperature, permeabilized in 0.5% Triton X-100 in PBS for 3 × 5 min, and incubated in blocking buffer (10% goat serum, 0.5% Triton X-100 in PBS) for 1 h. Coverslips were incubated with primary antibody in blocking buffer for 1 h at room temperature followed by washes in 0.5% Triton X-100 in PBS and incubation with secondary antibodies for 1 h. Coverslips were washed in PBS and mounted in mounting medium with DAPI (Vector Laboratories). For staining with the anti-LAMP1 antibody, 0.5% Triton X-100 was replaced by 0.05% saponin. Primary antibodies included mouse monoclonal anti-LAMP1 (1:200; Santa Cruz Biotechnology, 20011), rabbit polyclonal anti-β-coat protein (1:200; Thermo Fisher, PA1-061), rabbit polyclonal anti-transferrin receptor (1:100; Thermo Fisher, PA5–27739), and a mouse monoclonal anti-EGFR antibody against the extracellular domain of the EGFR (1:200; Santa Cruz Biotechnology, sc-120). Secondary antibodies included Alexa488-conjugated goat anti-mouse (1:2000; Invitrogen), Alexa594-conjugated goat anti-mouse (1:2000; Invitrogen), and Alexa594-conjugated goat anti-rabbit (1:2000; Invitrogen), respectively. To determine the localization of all GFP-tagged RAB protein variants in the absence of antibody staining, cells were fixed, briefly permeabilized in 0.5% Triton X-100 in PBS for 3 min, washed in PBS, and mounted as described above.

Images were acquired on a Leica TCS-SP5 confocal microscope using a 63× 1.4 numerical aperture oil UV objective (HCX PLAPO CS). Single excitation for each wavelength separately was used throughout all acquisitions (488 nm argon laser line and a 500–545 nm emission band pass; 543 HeNe laser line and a 556–673 nm emission band pass; 405 nm UV diode and a 422–466 nm emission band pass). The same laser settings and exposure times were used for image acquisition of individual experiments to be quantified. Ten to 15 image sections of selected areas were acquired with a step size of 0.4 μm, and z-stack images were analyzed and processed using Fiji.

### Alexa-EGF binding and uptake assays

Binding and uptake assays were performed essentially as described (24). Transfected HeLa cells were reseeded onto coverslips the day after transfection and serum-starved for 16 h. The following day, medium was replaced with fresh, serum-free medium containing 100 ng/ml Alexa555-EGF (Invitrogen) at 4 °C, which allows ligand binding to the receptor but prevents internalization. Alternatively, 100 ng/ml Alexa488-EGF (Invitrogen) was employed for experiments with siRNA-resistant or WT mRFP-RAB8A constructs. Control cells were washed twice with PBS followed by acid stripping (0.5 m NaCl, 0.2 m acetic acid, pH 2.5) for 3 min at 4 °C to confirm that labeled EGF was only surface-bound under those conditions. Upon binding, cells were washed twice with ice-cold PBS and transferred to prewarmed serum-free medium to allow uptake of bound Alexa555-EGF/Alexa488-EGF. At the indicated times, cells were fixed with 4% PFA in PBS for 15 min at room temperature and softly permeabilized with 0.5% Triton X-100 in PBS for 3 min before mounting with DAPI.

To measure the total number of Alexa555-EGF/Alexa488-EGF structures per cell, cells were circled, and a modified NIH Fiji macro (GFP-LC3 macro) was employed. At least 20 and up to 100 independent cells were analyzed for each condition and experiment, and analysis was done by an observer blind to conditions.

For intensity analyses, integrated densities of 30–50 cells per condition were determined, background-corrected, and normalized to 250 μm^2^ to correct for differences in cell size. Intensity per punctum was then determined by dividing the integrated density of the puncta by the number of puncta (71, 72). Analysis was performed using Fiji by an observer blind to conditions.

### Immunofluorescence-based EGFR recycling assays

Recycling assays were done as described previously (44). Briefly, transfected HeLa cells were seeded onto poly-l-lysine–coated coverslips the day after transfection and serum-starved overnight. The following day, cells were pretreated with 50 μg/ml cycloheximide (Calbiochem, 239765) in serum-free medium for 1 h at 37 °C to block novel protein synthesis. Cells were treated with 20 ng/ml nonlabeled EGF for 20 min at 4 °C in serum-free medium in the presence of cycloheximide to allow EGF binding to the receptor. Upon binding, cells were washed twice with ice-cold PBS, then transferred to prewarmed serum-free medium containing cycloheximide, and shifted to 37 °C for 10 min to allow for EGFR internalization (pulse). After the pulse, a mild acidic wash (pH 4.5) was performed on ice followed by two washes with ice-cold PBS. Subsequently, cells were incubated in serum-free medium containing cycloheximide at 37 °C to allow for EGFR recycling back to the plasma membrane, measured at different time points (chase; 15, 30, and 60 min). At the indicated times, cells were fixed with 4% PFA in PBS for 15 min followed by blocking with 10% goat serum in PBS and immunostaining with an EGFR antibody directed against the extracellular domain of the EGFR (1:200; Santa Cruz Biotechnology, sc-120) followed by staining with an Alexa488-coupled secondary antibody. Permeabilization was omitted from all steps to visualize only surface EGFR. For intensity quantifications, pictures were acquired the same day with the same settings, and analysis was performed with Fiji. Integrated densities of 30–50 cells per condition were determined, background-subtracted, and normalized to 250 μm^2^ to correct for differences in cell size.

### In vivo imaging and colocalization analysis

For live-cell fluorescence microscopy to determine colocalization of fluorescent EGF with the various RAB proteins in the absence or presence of pathogenic LRRK2, transfected cells were reseeded onto 35-mm glass-bottom dishes (ibidi) 24 h after transfection. For analysis of colocalization with fluorescent EGF, cells were serum-starved overnight. The next day, medium was replaced by phenol-free, serum-free DMEM (Gibco), and cells were incubated with 100 ng/ml Alexa647-EGF (Invitrogen) for 20 min at 4 °C to allow for fluorescent EGF surface binding. Subsequently, cells were washed twice in ice-cold PBS and incubated for 20 min at 37 °C to allow for internalization of bound EGF before image acquisition.

Cells were imaged on a Leica TCS-SP5 confocal microscope using a 63× 1.4 numerical aperture oil UV objective (HCX PLAPO CS) by acquiring individual z-stack images corresponding to the cell center. The JACoP plugin of Fiji was used for the quantification of colocalization of the different GFP-tagged RAB proteins with Alexa647-EGF. After thresholding, the percentage of colocalization was obtained by calculating the Manders' coefficients (M1 for red channel (Alexa647-EGF)), and the percentage of colocalization was obtained by M1 × 100 (24). A total number of 15–20 independent cells were analyzed per condition per experiment.

### Cell extracts and Western blotting

Cells were collected 48 h after transfection, washed in PBS, and resuspended in cell lysis buffer (1% SDS in PBS containing 1 mm PMSF, 1 mm Na_3_VO_4_, and 5 mm NaF). Extracts were sonicated, boiled, and centrifuged at 13,500 rpm for 10 min at 4 °C. Protein concentration of supernatants was estimated using the BCA assay (Pierce), and extracts were immediately resolved by SDS-PAGE, transferred to nitrocellulose membranes, and probed with primary antibodies overnight at 4 °C. Antibodies used for immunoblotting included a rabbit polyclonal anti-GFP (1:2000; Abcam, ab6556), a mouse monoclonal anti-GAPDH (1:2000; Abcam, ab9484), a mouse monoclonal anti-LRRK2 (1:1000; NeuroMab, 75-253), a mouse monoclonal anti-RAB8A antibody (1:1000; BD Biosciences, 610844), a mouse monoclonal anti-tubulin antibody (clone DM1A; 1:10,000; Sigma), a sheep polyclonal anti-RAB8A antibody (1:500; S969D, MRC-PPU Reagents), a rabbit polyclonal anti-RAB7 antibody (1:1000; Sigma, R4779), a mouse monoclonal anti-RAB11 antibody (1:1000; BD Biosciences, 610656), and a knockout-validated rabbit monoclonal anti-RAB4 antibody (1:1000; Abcam, ab109009). Membranes were washed and incubated with secondary antibodies (anti-rabbit HRP-conjugated antibody (1:2000; Dako Cytomation) or anti-mouse HRP-conjugated antibody (1:2000; Dako Cytomation)) for 60 min at room temperature followed by detection using ECL reagents (Roche Diagnostic GmbH). A series of timed exposures were undertaken to ensure that densitometric analyses were performed at exposures within the linear range, films were scanned, and densitometric analysis was performed using Quantity One (Bio-Rad).

### Determination of EGFR steady-state levels and EGFR degradation

For determination of steady-state levels and EGFR degradation, we employed HEK293T cells because overexpression and transfection efficiencies are higher than in HeLa cells (24). HEK293T cells were cultured and transfected overnight using 6 μl of lipoD reagent (SignaGen Laboratories, SL100688) and 2 μg of FLAG-tagged LRRK2 DNA (300 ng for pCMV) as described previously (39). The following day, cells were split into poly-l-lysine–coated 6-well plates and processed 48 h after transfection. For determination of steady-state levels of endogenous EGFR, cells were washed in ice-cold PBS and collected by scraping and mechanical disruption. Cells were centrifuged at 5000 rpm for 2 min at 4 °C, and the cell pellet was resuspended in 100 μl of lysis buffer (10% SDS and 1 mm PMSF in PBS). Samples were sonicated and centrifuged at 10,000 rpm for 10 min at 4 °C, and supernatants (25 μg of total protein) were analyzed by SDS-PAGE and Western blotting using a mouse monoclonal anti-FLAG antibody (1:500; Sigma, F1804), a rabbit monoclonal anti-EGFR antibody (1:1000; Cell Signaling Technology, D38B1), or a mouse monoclonal anti-tubulin antibody (clone DM1A; 1:10,000; Sigma) as a loading control.

For determination of EGFR degradation, HEK293T cells were transfected and plated onto poly-l-lysine–coated 6-well plates, and degradation assays were performed 48 h after transfection. Cells were serum-starved for 1 h in the presence of 1 μg/ml cycloheximide followed by incubation with 100 ng/ml nonlabeled EGF (Sigma, E9644) for 20 min at 4 °C in serum-free medium containing cycloheximide. After washing in ice-cold PBS, time point 0 was collected by cell scraping. For the remaining time points, cells were incubated in serum-free medium containing cycloheximide at 37 °C for distinct periods of time (30, 60, and 90 min) followed by cell scraping, and cell extracts were prepared and analyzed by SDS-PAGE and Western blotting as described above.

### GST-RILP pulldown assays and active RAB7 determination

GST-RILP pulldown assays were essentially performed as described (24, 42). Briefly, GST-RILP vector was transformed into *Escherichia coli* strain BL21, and 250 ml of LB was inoculated with a 1-ml overnight culture grown at 37 °C to an OD of 0.6–0.8. Isopropyl 1-thio-β-d-galactopyranoside (EMD Biosciences) (0.5 mm) was added, and bacteria were induced for protein production for 3–4 h at 28 °C. Bacterial cells were pelleted and washed with cold PBS, and cell pellets were frozen at −20 °C. Pellets were resuspended in 5 ml of ice-cold purification buffer (25 mm Tris-HCl, pH 7.4, 150 mm NaCl, 0.5 mm EDTA, 1 mm DTT, 0.1% Triton X-100, and 1 mm PMSF), and lysates were sonicated and cleared by centrifugation. Supernatant was diluted with another 5 ml of ice-cold purification buffer, and GST-RILP was purified using 300 μl of a pre-equilibrated 50% slurry of GSH-Sepharose 4B beads (GE Healthcare) and incubation for 1 h at 4 °C on a rotary wheel. Beads were washed with purification buffer, resuspended to a 50% slurry, and kept at 4 °C. A sample (5 μl) was separated by SDS-PAGE and analyzed by Coomassie Brilliant Blue staining to determine protein purity, and protein concentration was estimated by BCA assay (Pierce). Beads were used with cell lysates within 2 days of preparation. Transfected HEK293T cells (one 10-cm-diameter dish per assay) were collected by centrifugation, washed in PBS, and resuspended in pulldown buffer (20 mm HEPES, pH 7.4, 100 mm NaCl, 5 mm MgCl_2_, 1% Triton X-100, and 1 mm PMSF), and lysates were cleared by centrifugation at 13,500 rpm for 10 min at 4 °C. GST-RILP pulldown assays were performed in 1 ml of pulldown buffer containing 300 μg of cell lysate and 60 μl of 50% slurry beads pre-equilibrated in pulldown buffer. Beads were incubated on a rotary wheel overnight at 4 °C and washed twice with ice-cold pulldown buffer, and bound proteins were eluted by adding 40 μl of 1× sample buffer/β-mercaptoethanol and boiling for 4 min at 95 °C prior to separation by SDS-PAGE.

Alternatively, active RAB7 levels were determined with a RAB7 activation assay kit (NewEast Biosciences) according to the manufacturer's specifications and as described previously (43). Briefly, cells were transfected as described above, and lysates (2 mg of total protein) were immunoprecipitated with a conformation-specific anti-RAB7-GTP mouse mAb (NewEast Biosciences, 26923) bound to protein A/G–agarose, and the precipitated RAB7-GTP was detected by immunoblotting with a nonconformation-specific rabbit polyclonal anti-RAB7 antibody (1:1000; Sigma, R4779). As a positive control, extracts were incubated with 100 μm GTPγS (NewEast Biosciences, 30302) for 30 min at 30 °C to activate all available RAB7 prior to immunoprecipitation.

### Cell culture, transient transfection, and protein purification

HEK-293T cells were maintained in DMEM supplemented with 10% fetal bovine serum and 1× penicillin/streptomycin at 37 °C and in a 5% CO_2_ atmosphere. Cells were transfected with the 3XFLAG-RABs or with WT or G2019S 3XFLAG-LRRK2 plasmid DNAs using polyethylenimine (PEI) with a DNA:PEI ratio of 1:2 (w/w). Cells were harvested 72 h post-transfection for biochemical assays.

Cells were lysed in buffer A (20 mm Tris-HCl, pH 7.5, 150 mm NaCl, 1 mm EDTA, 2.5 mm Na_4_O_7_P_2_, 1 mm β-glycerophosphate, 1 mm NaVO_4_, 0.5% Tween 20, protease inhibitor mixture, and 1×Complete Mini protease inhibitor mixture (Roche Applied Sciences)). Cleared lysates (1 ml) were incubated with anti-FLAG-M2–agarose beads (Sigma) by rotating overnight at 4 °C. Resin complexes were washed with different buffers (twice with 20 mm Tris-HCl, 500 mm NaCl, and 0.5% Tween 20; twice with 20 mm Tris-HCl, 300 mm NaCl, and 0.5% Tween 20; twice with 20 mm Tris-HCl, 150 mm NaCl, and 0.5% Tween 20; twice with 20 mm Tris-HCl, 150 mm NaCl, and 0.1% Tween 20; and twice with 20 mm Tris-HCl, 150 mm NaCl, and 0.02% Tween 20), and proteins were eluted in kinase buffer (25 mm Tris-HCl, pH 7.5, 5 mm β-glycerophosphate, 2 mm DTT, 0.1 mm Na_3_VO_4_, 10 mm MgCl_2_, 0.02% Tween 20, and 150 ng/μl 3XFLAG peptide) for 45 min at 4 °C with shaking. Eluted proteins were resolved by SDS-PAGE and stained with Coomassie G-250 to verify protein purity. RAB and LRRK2 protein concentrations were estimated by densitometry against a standard curve of increasing concentrations of bovine serum albumin (BSA) (20, 50, 100, and 150 ng/μl).

### Phosphorylation assays

Phosphorylation reactions were performed either in the presence or absence of 1 μm LRRK2 kinase inhibitor PF 06447475 (Tocris Bioscience). Reactions were performed in kinase buffer in the presence of 30 nm LRRK2 and a 300 nm concentration of the respective RAB proteins and with [γ-^33^P]ATP (1 μCi/reaction; PerkinElmer Life Sciences) and 200 μm cold ATP (Sigma-Aldrich) at 30 °C for 1 h in a final volume of 30 μl. Reactions were terminated with 4× Laemmli buffer and boiling at 95 °C for 10 min. Phosphorylation reaction samples were resolved on precast 4–20% SDS-polyacrylamide gels (Bio-Rad) and transferred onto PVDF membranes. Incorporated radioactivity was detected by a phosphor screen and Cyclone acquisition (PerkinElmer Life Sciences), and the same membranes were probed with HRP-conjugated anti-FLAG antibody to confirm equal protein loading.

### Statistical analysis

All data are expressed as means ± S.E. Data were analyzed by one-way analysis of variance with Tukey's post hoc test, and *p* < 0.05 was considered significant.

## Author contributions

P. R.-R., A. P. T., and S. H. conceptualization; P. R.-R. data curation; P. R.-R. formal analysis; P. R.-R. and S. H. validation; P. R.-R., M. R.-L., J. M.-P., A. B., and E. G. investigation; P. R.-R. visualization; P. R.-R. and S. H. writing-original draft; P. R.-R., M. R.-L., J. M.-P., A. P. T., A. B., E. G., and S. H. writing-review and editing; S. H. resources; S. H. supervision; S. H. funding acquisition; S. H. methodology; S. H. project administration.

## Supplementary Material

Supporting Information

## Footnotes

3 The abbreviations used are:

LRRK2: leucine-rich repeat kinase 2
PD: Parkinson's disease
EGF: epidermal growth factor
EGFR: epidermal growth factor receptor
GDI: GDP dissociation inhibitor
GEF: guanine nucleotide exchange factor
RILP: RAB-interacting lysosomal protein
Alexa: Alexa Fluor
mRFP: monomeric red fluorescent protein
GAP: GTPase-activating protein
DMEM: Dulbecco's modified Eagle's medium
PFA: paraformaldehyde
DAPI: 4′,6-diamidino-2-phenylindole
PMSF: phenylmethylsulfonyl fluoride
HRP: horseradish peroxidase
GTPγS: guanosine 5′-O-(gamma-thio)-triphosphate
PEI: polyethylenimine
ctrl: control
GAPDH: glyceraldehyde-3-phosphate dehydrogenase.
