# Supplementary material for: The G2019S variant of leucine-rich repeat kinase 2 (LRRK2) alters endolysosomal trafficking by impairing the function of the GTPase RAB8A
Source: J Biol Chem. 2019 Feb 1;294(13):4738–58. doi: 10.1074/jbc.RA118.005008 (PMC6442034; doi:10.1074/jbc.RA118.005008)
Supplement: Supporting Information [file supp_294_13_4738__index.html]

The G2019S variant of leucine-rich repeat kinase 2 (LRRK2) alters endolysosomal trafficking by impairing the function of the GTPase RAB8A — G2019S LRRK2 impairs RAB8A function — The G2019S variant of leucine-rich repeat kinase 2 (LRRK2) alters endolysosomal trafficking by impairing the function of the GTPase RAB8A — G2019S LRRK2 impairs RAB8A function — Supporting Information 

# The G2019S variant of leucine-rich repeat kinase 2 (LRRK2) alters endolysosomal trafficking by impairing the function of the GTPase RAB8A

## Supporting Information

- Supporting Information (to be published online) - supplemental file
