# Supplementary material for: The G2019S variant of leucine-rich repeat kinase 2 (LRRK2) alters endolysosomal trafficking by impairing the function of the GTPase RAB8A
Source: J Biol Chem. 2019 Feb 1;294(13):4738–58. doi: 10.1074/jbc.RA118.005008 (PMC6442034; doi:10.1074/jbc.RA118.005008)
Supplement: Supporting Information [file supp_RA118.005008_139592_2_supp_277358_pm4kbz.pdf]

Supplementary Figures

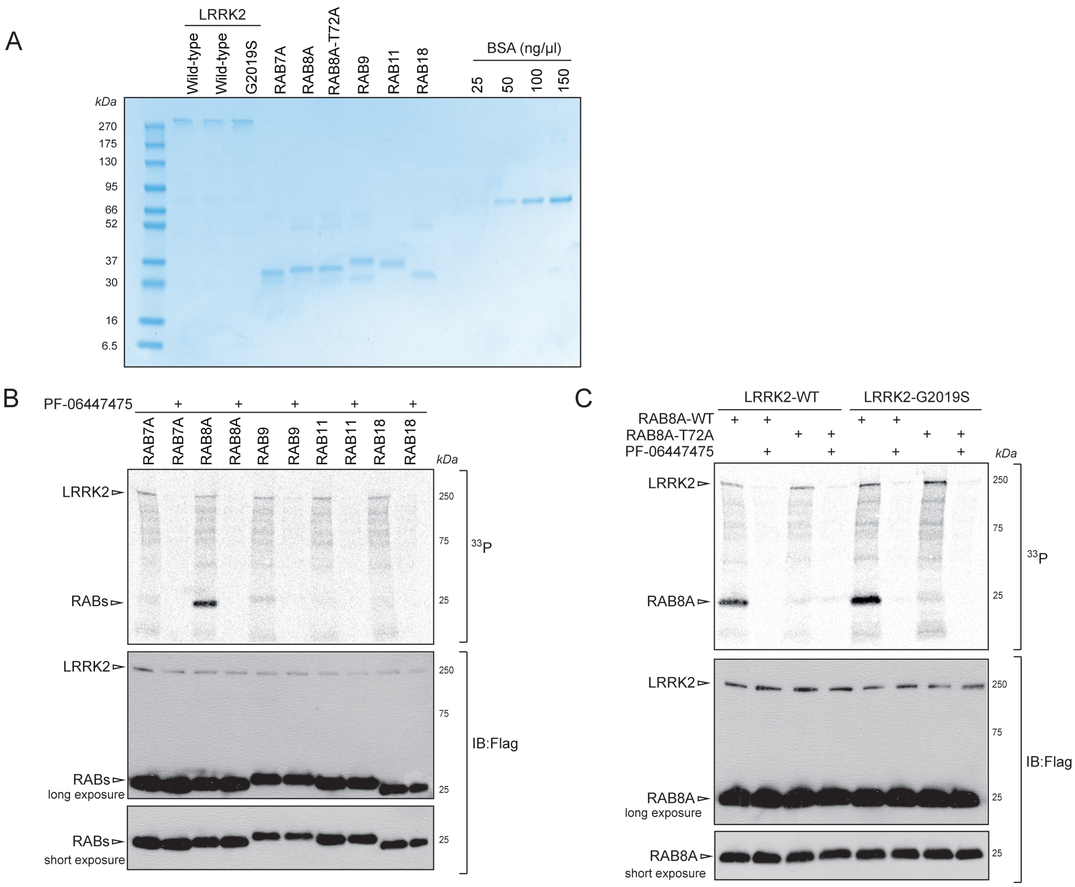

Fig. S1. LRRK2 phosphorylates RAB8A but not endolysosomal RAB7A/RAB9 proteins. (**A**) Representative Coomassie blue stained SDS-PAGE of purified 3x-flag-tagged LRRK2 or 3x-flag-tagged RAB proteins after 3xflag peptide elution. Protein concentration was estimated by a calibration curve with BSA standards of known concentrations. (**B**) *In vitro* radioactive kinase assays of 3x-flag-tagged wildtype LRRK2 and 3x-flag-tagged RAB7A, RAB8A, RAB9, RAB11 or RAB18, purified from

HEK293T cells (1:10 ratio of LRRK2:Rab proteins). Incorporated radioactivity was revealed by phosphoscreen (upper panel), and total protein loading by anti-flag (IB:Flag) immunoblotting (lower panel). LRRK2 kinase inhibitor PF 06447475 was used at 1 μM to confirm LRRK2-specific phosphorylation. (**C**) *In vitro* radioactive kinase assays of 3x-flag-tagged wildtype or G2019S LRRK2 in the presence of either 3x-flag-tagged wildtype RAB8A or phosphodeficient RAB8A-T72A as indicated. Incorporated radioactivity was revealed by phosphoscreen (upper panel), and total protein loading by anti-flag (IB:Flag) immunoblotting (lower panel). LRRK2 kinase inhibitor PF 06447475 was used at 1 μM to confirm LRRK2-specific phosphorylation.

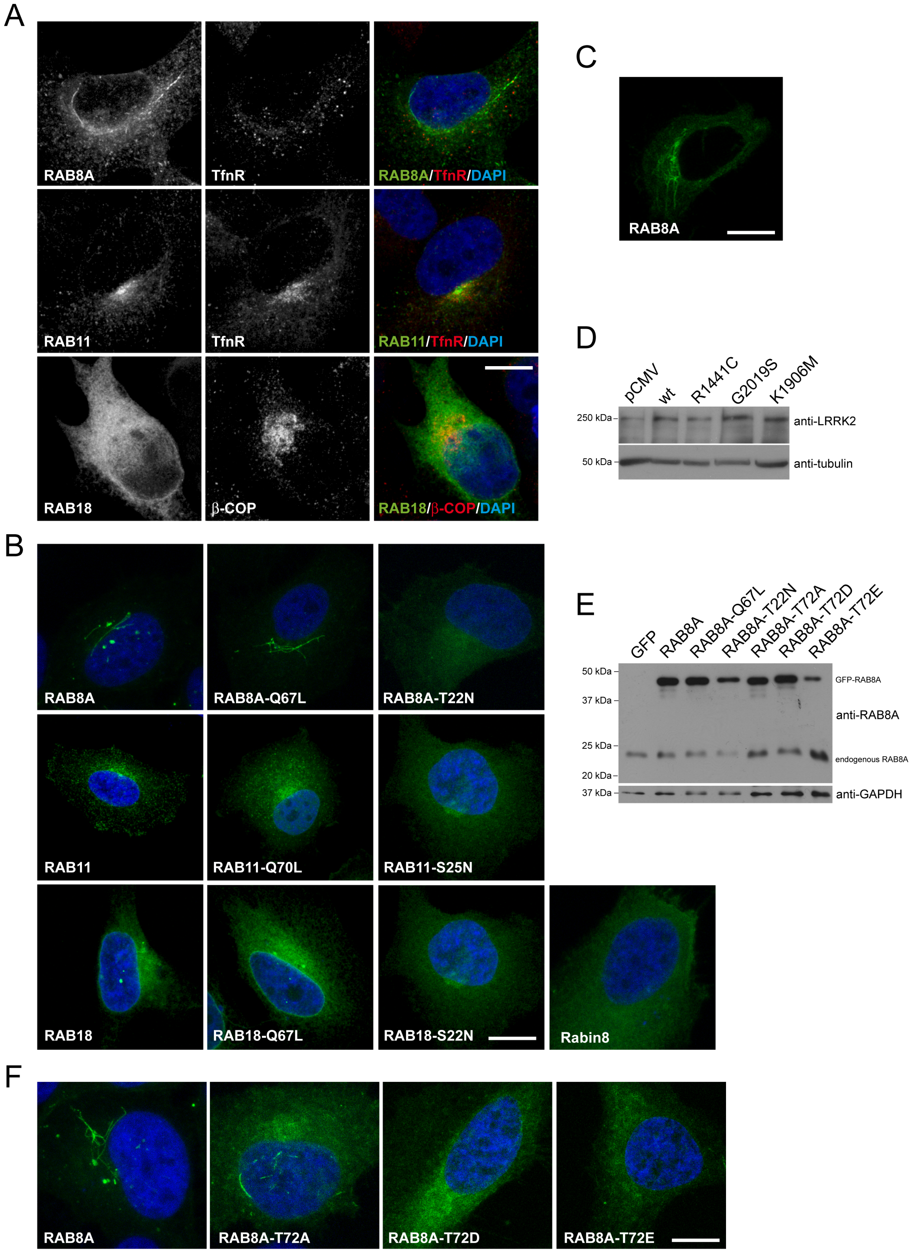

Fig. S2. Subcellular localization and expression levels of different RAB proteins. (**A**) HeLa cells were transfected with GFP-tagged RAB constructs as indicated, and stained with antibodies against a marker for endolysosomes (LAMP1), endocytic recycling compartment (TfnR) or trans-Golgi (β-COP). Scale bar, 10 μm. (**B**) HeLa cells were transfected with GFP-tagged RAB constructs as indicated. To maintain proper membrane

association of the different RAB proteins, cells were fixed but only briefly permeabilized before mounting with DAPI. Scale bar, 10 μm. (**C**) Individual z-stack of live HeLa cell transfected with GFP-RAB8A. Scale bar, 10 μm. (**D**) HeLa cells were transfected with either empty vector (pCMV) or the indicated GFP-tagged LRRK2 constructs, and cell extracts (30 μg) analyzed by Western blotting for LRRK2 protein levels, and for tubulin as loading control. (**E**) HeLa cells were transfected with either GFP, or GFP-tagged RAB8A constructs as indicated, and cell extracts (30 μg) analyzed by Western blotting for RAB8A, and for GAPDH as loading control. (**F**) Example of HeLa cells transfected with the indicated GFP-tagged RAB8A variants, fixed and mounted with DAPI (blue). Scale bar, 10 μm.

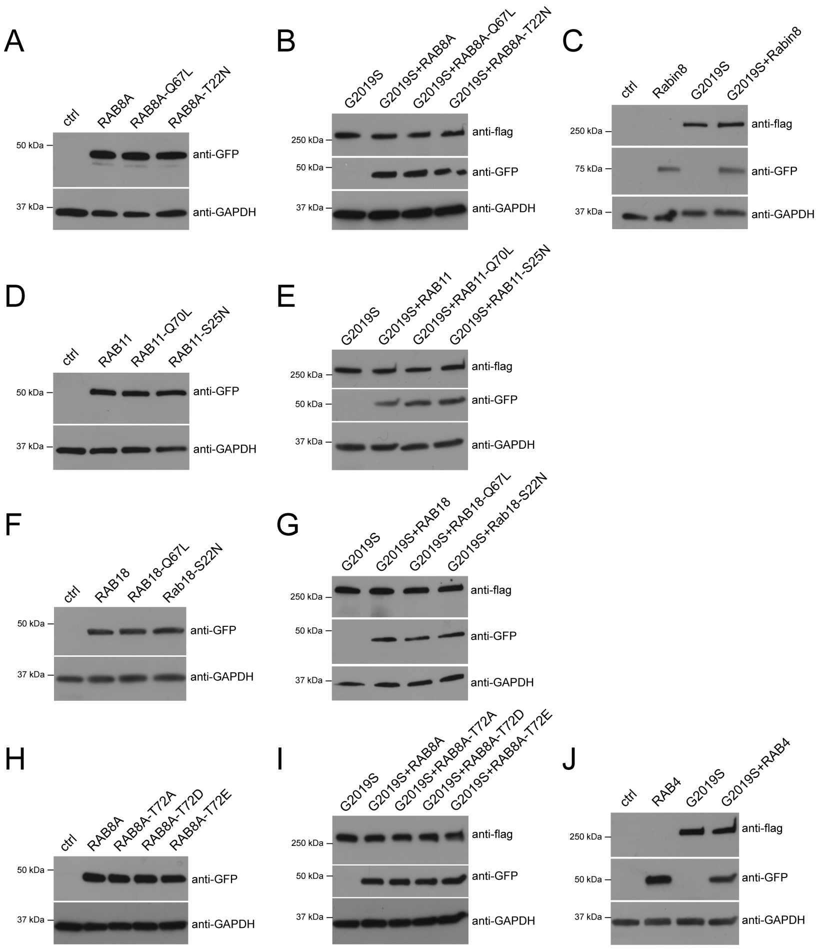

Fig. S3. Expression and co-expression levels of various RAB proteins with G2019S LRRK2. (**A**) HeLa cells were transfected with GFP-tagged RAB8A constructs as indicated, and cell extracts (30 μg) analyzed by Western blotting for GFP, and for GAPDH as loading control. (**B**) HeLa cells were transfected with flag-G2019S along with the indicated GFP-tagged RAB8A constructs as indicated, and cell extracts (30 μg) analyzed by Western blotting for flag, GFP, and for GAPDH as loading control. (**C**) HeLa cells were transfected with GFP-tagged Rabin8, flag-G2019S, or co-transfected as indicated, and cell extracts (30 μg) analyzed by Western blotting for flag, GFP, and for GAPDH as loading control. (**D**) Same as (A), but cells transfected with GFP-tagged

RAB11 constructs as indicated. (**E**) Same as (B), but cells co-transfected with flag-G2019S and the GFP-tagged RAB11 constructs as indicated. (**F**) Same as (A), but cells transfected with GFP-tagged RAB18 constructs as indicated. (**G**) Same as (B), but cells co-transfected with flag-G2019S and GFP-tagged RAB18 constructs as indicated. (**H**) Same as (A), but cells transfected with GFP-tagged RAB8A constructs as indicated. (**I**) Same as (B), but cells co-transfected with flag-G2019S and GFP-tagged RAB8A constructs as indicated. (**J**) Cells were transfected with GFP-tagged RAB4, flag-G2019S, or co-transfected as indicated, and cell extracts (30 μg) analyzed by Western blotting for flag, GFP, and for GAPDH as loading control.

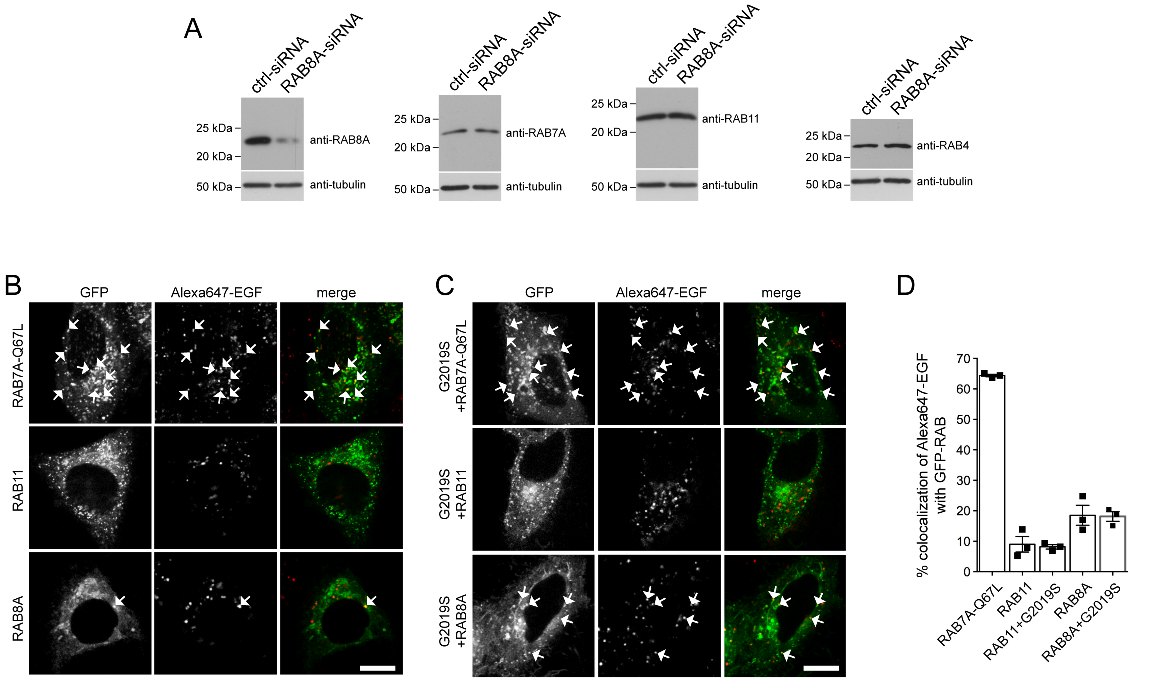

Fig. S4. Specificity of RAB8A knockdown, and lack of Alexa647-EGF accumulation in RAB8A- or RAB11-positive compartments. (**A**) HeLa cells were treated with ctrl-siRNA or RAB8A-siRNA, and extracts subjected to Western blotting against various RAB proteins as indicated. (**B**) Cells were transfected with GFP-tagged RAB proteins as indicated, loaded with Alexa647-EGF for 20 min and followed by live imaging. Arrows indicate colocalization. Scale bar, 10 μm. (**C**) Cells were transfected with GFP-tagged RAB proteins and G2019S LRRK2 as indicated, loaded with Alexa647-EGF for 20 min followed by live imaging. Arrows indicate colocalization. Scale bar, 10 μm. (**D**) Quantification of colocalization of Alexa647-EGF with GFP-tagged RAB proteins in the absence or presence of G2019S LRRK2 as indicated (Manders coefficient 1 x 100). N=3 independent experiments.

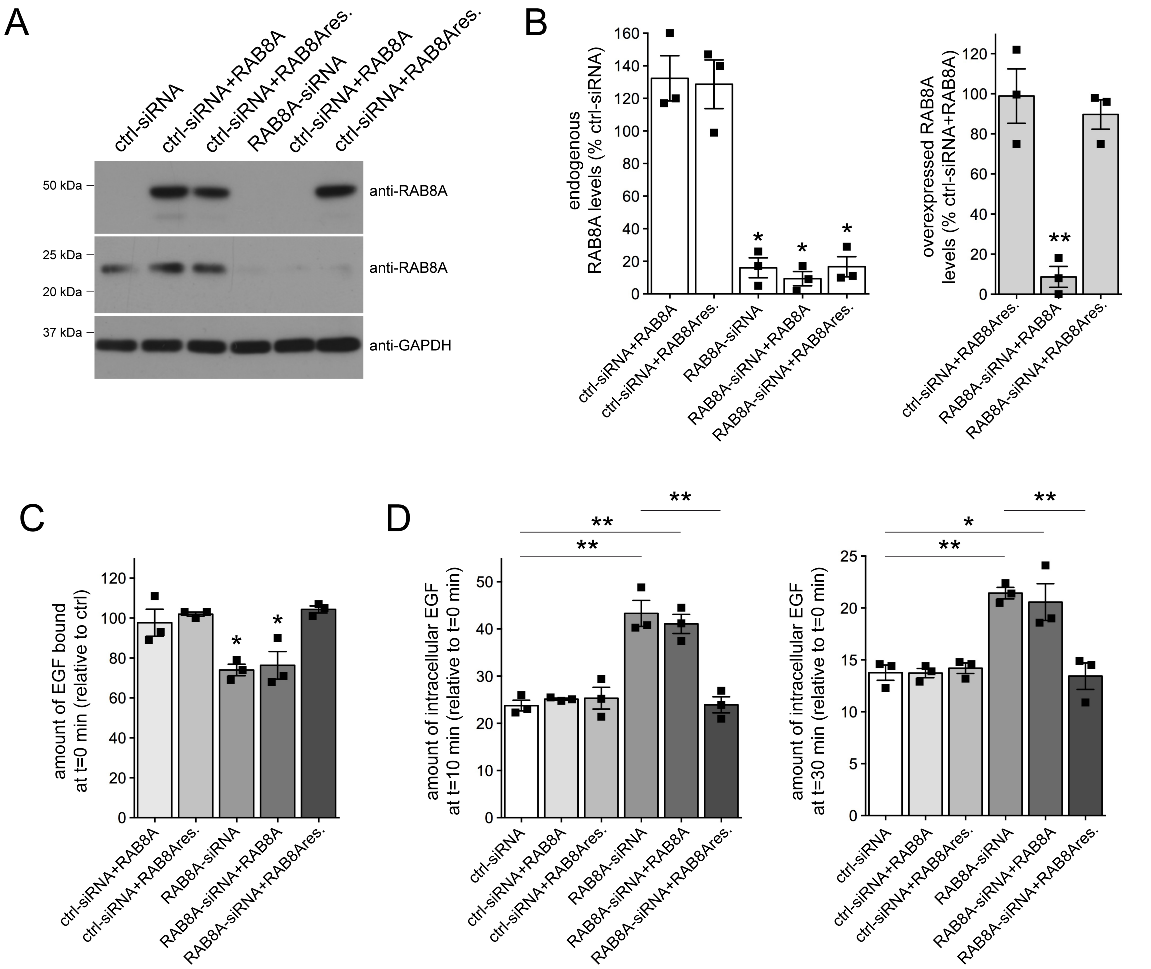

Fig. S5. siRNA-resistant RAB8A variant rescues the G2019S LRRK2-mediated endolysosomal trafficking deficits. (**A**) HeLa cells were either transfected with ctrl-siRNA or RAB8A-siRNA, and either with wildtype mRFP-RAB8A (RAB8A) or an siRNA-resistant version of mRFP-RAB8A (RAB8res.), and cell extracts (30 μg) analyzed by Western blotting for mRFP-RAB8A levels, endogenous RAB8A levels and GAPDH as loading control. (**B**) Quantification of the type of experiments depicted in (A). Endogenous RAB8A levels (left) were normalized to levels in the presence of ctrl-siRNA, and overexpressed mRFP-RAB8A levels (right) normalized to levels in the presence of ctrl-siRNA and wildtype RAB8A (RAB8A). N=3 independent experiments.

*P < 0.05; **P < 0.01. (**C**) Cells were either transfected with ctrl-siRNA or RAB8A-siRNA in the presence or absence of wildtype or siRNA-resistant mRFP-RAB8A as indicated, and surface-bound fluorescent Alexa488-EGF quantified. N=3 independent experiments. *P < 0.05. (**D**) Cells were either transfected with ctrl-siRNA or RAB8A-siRNA in the presence or absence of wildtype or siRNA-resistant mRFP-RAB8A as indicated, followed by quantification of internalized fluorescent Alexa488-EGF at 10 min (left) and 30 min (right). N=3 independent experiments. * P < 0.05; ** P < 0.01.
